# Risk factors for the intra-individual double burdens of malnutrition among reproductive-age women in India: a secondary analysis of three rounds of the National Family and Health Survey

**DOI:** 10.1136/bmjph-2025-002730

**Published:** 2025-10-23

**Authors:** Hannah Gardner, Vani Sethi, Tuck Seng Cheng, Manisha Nair

**Affiliations:** 1 Nuffield Department of Population Health, University of Oxford, Oxford, UK; 2 UNICEF Regional Office for South Asia, Kathmandu, Nepal

**Keywords:** Nutrition Surveys, Body Mass Index, Female, Epidemiology, Obesity

## Abstract

**Introduction:**

Underweight, overweight/obesity and anaemia are prevalent among reproductive-age women in India, but factors affecting their intra-individual co-occurrence are unclear. Our objectives were to examine the prevalence, spatial distribution and risk factors for concurrent ‘double burdens’ of anaemia with underweight or overweight/obesity within the same individual.

**Methods:**

Using data from reproductive age women (15–49 years) in the Indian National Family Health Surveys 2005–2006, 2015–2016 and 2019–2021, we calculated the national prevalence of the intra-individual double burdens of malnutrition and mapped their district-level distributions. We examined the association of 28 potential risk factors with anaemia, underweight, overweight/obesity and their co-occurrence using multilevel logistic regression and calculated area under the curve (AUC) statistics.

**Results:**

The underweight-anaemia double burden affected 11%, and overweight/obesity-anaemia double burden affected 21% of women in 2019–2021. Overweight/obesity-anaemia was prevalent in northern, southern and coastal districts, while underweight-anaemia was most prevalent in central India. For underweight-anaemia, important risk factors were tobacco consumption, younger age, lower education, lower household wealth, scheduled tribe background, larger household size, region and living in a high malaria transmission area. For overweight/obesity-anaemia, risk factors included older age, being educated, higher household wealth, region and living in an area with more hot days than average in recent years. AUC statistics showed a significant role of environmental factors.

**Conclusions:**

The double burdens present significant public health challenges for India. The spatial and sociodemographic distinctness of the burdens suggests the possibility of evidence-informed targeting of programmes.

**WHAT IS ALREADY KNOWN ON THIS TOPIC**

- Anaemia, underweight and overweight/obesity are all highly prevalent among women of reproductive age in India and are increasingly found to co-occur within individuals.
- Existing studies have highlighted spatial patterns and broad sociodemographic correlates, particularly for overweight/obesity-anaemia. However, evidence remains limited on risk factors beyond sociodemographics and on the underweight-anaemia double burden.

**WHAT THIS STUDY ADDS**

- This study presents a consolidated and comprehensive analysis of risk factors for both underweight-anaemia and overweight/obesity-anaemia burdens.
- It assesses a wide range of diet/lifestyle, service access, sociodemographic and environmental variables; maps double burden prevalence at the district level; and considers changes in risk factor effect sizes over time.

**HOW THIS STUDY MIGHT AFFECT RESEARCH, POLICY AND PRACTICE**

- Findings suggest that the two double burden types have distinct sociodemographic and spatial patterns. This supports the potential value of targeted strategies—by geography or risk group—in India’s malnutrition programmes.

## Introduction

 The WHO has called for the public health challenges of overweight and obesity to be considered alongside those of underweight and micronutrient deficiency, in recognition that these different forms of malnutrition increasingly co-occur within populations.^1 2^ India concurrently has the largest burden of reproductive-age women with underweight or anaemia in the world^3 4^ and the third largest burden of women with overweight/obesity.^5^ In this context, the co-occurrence of multiple forms of malnutrition within an individual is increasingly a public health concern. Co-occurrence may compound the risks of poor maternal outcomes,^6^,^8^ weakened immune function^9^,^11^ and the intergenerational transfer of malnutrition,^12 13^ which are common to all three forms of malnutrition.

Anaemia—a low number of red blood cells or low concentration of haemoglobin—is a multifactorial condition with aetiologies which include nutritional deficiency, infection, chronic disease and inflammation, red blood cell disorders, and gynaecological and obstetric conditions.^14^ The frequent co-occurrence of anaemia and underweight in individuals can be partly explained by shared determinants of poor nutritional intake and increased susceptibility to infections. Emerging evidence also suggests biological pathways linking overweight and obesity with anaemia: chronic inflammation associated with excess body weight may elevate hepcidin levels, thereby reducing iron absorption and increasing the risk of iron deficiency anaemia.^15^ The relationship may be bidirectional, as symptoms and conditions commonly associated with anaemia—such as fatigue, reduced appetite and altered thyroid function—can contribute to either weight loss or weight gain.^1^ These interlinked mechanisms across different forms of malnutrition render it important to understand drivers of co-occurrence.

The double burden of malnutrition has multiple operational definitions,^16^ but here, we use it to specify the co-occurrence of anaemia with either underweight or overweight/obesity in the same individual. Double burdens of malnutrition at both the population and individual level have increasingly been studied in many low- and middle-income countries such as India, where high levels of undernutrition persist, but the prevalence of overweight and obesity has risen sharply. Previous research in India has reported the high prevalence of the double burden of overweight/obesity-anaemia^17^,^20^ and identified significant spatial clusters of high anaemia and high overweight/obesity in districts in Gujarat, Tamil Nadu, Andhra Pradesh and certain northern states.^21^ Certain studies have used data from three rounds of India’s National Family and Health Survey (NFHS) and shown that the prevalence of underweight-anaemia has almost halved between 2005–2006 and 2019–2021, while the prevalence of overweight/obesity-anaemia more than doubled in the same period.^17 18^ However, these studies have used international thresholds for defining overweight and obesity, whereas evidence suggests that Asian individuals have the same proportion of body fat and experience the same risks of cardiometabolic diseases at lower levels of BMI compared with non-Asian populations.^22^,^24^ Using international cut-thresholds risks underestimating overweight/obesity and its double burden among women in India.

Studies in India have found consistent associations between the overweight-anaemia double burden and older age, urban residence, and higher wealth and education. However, studies rarely examined risk factors beyond key sociodemographics.^1820 21 25^,^29^ This leaves an evidence gap for the role of modifiable factors such as nutritional intake and access to health and social services as potential policy targets. To the best of the authors’ knowledge, no studies in India have assessed risk factors for the co-occurrence of anaemia and underweight. Studies investigating overweight/obesity-anaemia have excluded underweight individuals or included them in the baseline group in analyses, which may obscure relationships in the data if the association between weight categories and anaemia is U-shaped. There is a need for a comprehensive assessment of risk factors for both double burden and single burden malnutrition outcomes, using individuals without malnutrition (no anaemia and normal weight) as the baseline. As the prevalence of anaemia and underweight has changed substantially since 2005–2006, it is likely that the characteristics and risk factors for the single and double burdens of malnutrition have also shifted. While one previous study assesses risk factors for overweight/obesity-anaemia in three NFHS survey rounds, significant differences in risk factor effect sizes across years were not investigated.^18^

This study aims to address these research gaps and present a comprehensive, consolidated analysis of the intra-individual co-occurrence of anaemia, underweight and overweight/obesity among Indian women. While much research on the risk factors for malnutrition has focused on pregnant women and children under 5 years of age due to particular risks for maternal complications, preterm birth and child development,^13 30 31^ malnutrition has effects across the life course on bone and muscle function, work productivity, and infectious or chronic disease risk.^32^,^34^ Recent research also highlights the importance of preconception nutrition for many maternal and child health outcomes.^35 36^ This analysis focuses on malnutrition in all reproductive-age women aged 15–49 years.

The primary objectives of this study were to: (i) estimate the prevalence of the underweight-anaemia and overweight/obesity-anaemia double burdens among reproductive-age women in India and map their spatial distributions at the district level and (ii) assess a comprehensive range of potential risk factors for associations with the two double burdens. Secondary objectives were to (i) estimate the change in prevalence of underweight-anaemia and overweight/obesity-anaemia over time and (ii) assess whether associations of the two double burdens with potential risk factors had changed over time.

## Methods

### Data source and study design

This study is a secondary analysis of data from the National Family Health Survey (NFHS), rounds 3 (2005–2006),^37^ 4 (2015–2016)^38^ and 5 (2019–2021).^39^ The NFHS is the Demographic and Health Survey (DHS) for India and uses a two-stage stratified sampling design whereby households are sampled from selected clusters (a village or census enumeration block). The sample is designed to be representative at both the state (NFHS rounds 3, 4 and 5) and district (NFHS rounds 4 and 5 only) level. Every woman aged 15–49 years who slept in a selected dwelling on the previous night is eligible for interview. Detailed explanation of the study design is reported elsewhere.^40^

### Study population

Women aged 15–49 years with valid measurements of both body mass index (BMI) and haemoglobin concentration who were neither pregnant nor fewer than 3 months post-partum were eligible for initial inclusion for estimating prevalence.

A further inclusion criterion for risk factor analysis specified that women were usual (‘de jure’) residents of the interviewed household, as analysis included household-level variables.

Differences in the sociodemographic characteristics of included and excluded participants for both initial and further inclusion criteria are presented in online supplemental Table 1.

### Outcome variables

Women with haemoglobin levels under 12.0 g/dL were defined as having anaemia, as is consistent with the WHO definition for non-pregnant women.^41^ The WHO’s 2024 updated recommendations^42^ for adjustments to haemoglobin levels according to altitude and smoking status were used, as opposed to the DHS standard procedure which followed older recommendations.^43^

Asian regional cut-offs were used to determine BMI categorisation for individuals aged 18 and older (underweight, BMI<18.5 kg/m^2^; overweight/obesity, BMI ≥23 kg/m^2^). For individuals between ages 15 and 17 inclusive, cut-offs corresponding to these BMI values at age 18 were calculated using the formula provided by Cole and Lobstein^44^ based on International Obesity Task Force growth reference curves (online supplemental Table 3).

**Figure 1:**
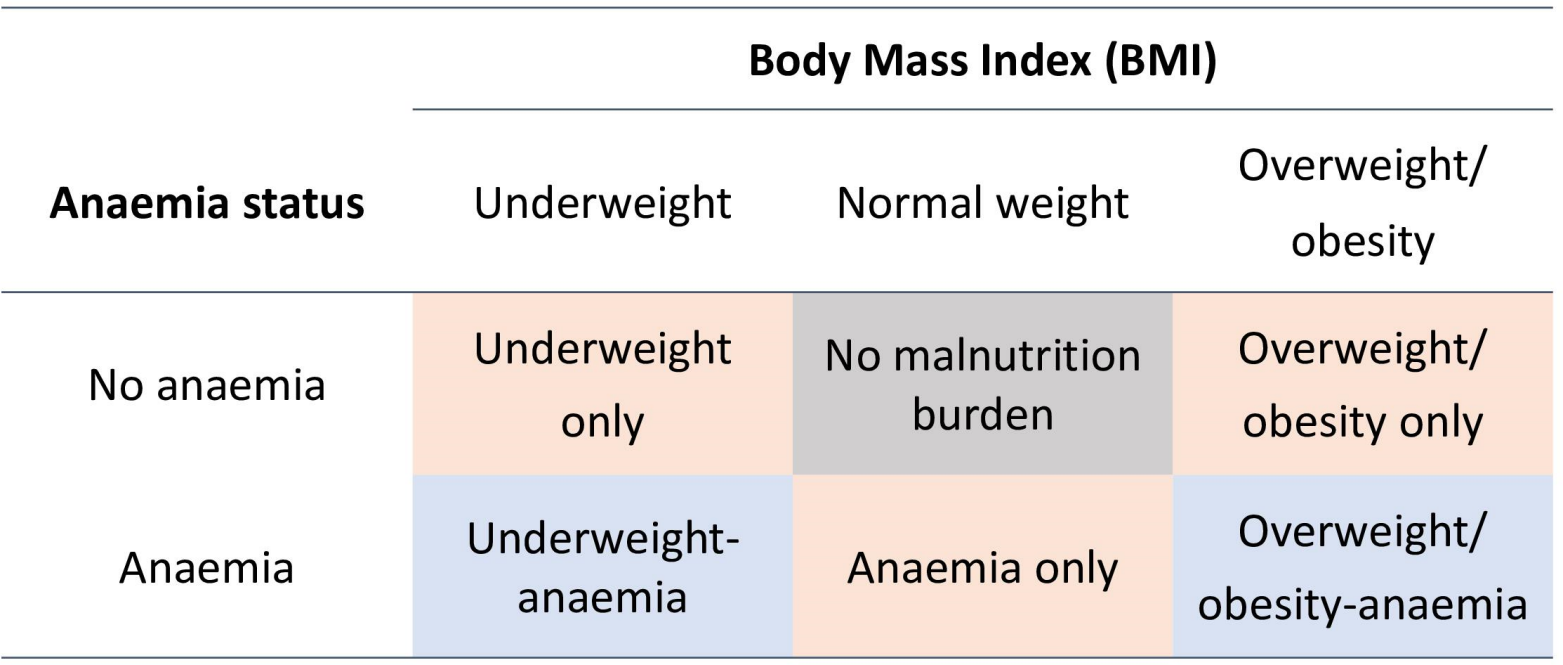
Outcome categories used in analysis. Notes: Categories are mutually exclusive and exhaustive, dividing all women of reproductive age. ‘No malnutrition burden’ outcome is shaded in grey. ‘Single burden’ outcomes are shaded in orange. ‘Double burden’ outcomes are shaded in blue. Anaemia: haemoglobin concentration <12.0 g/dL. Underweight: BMI <18.5 kg/m^2^. Overweight/obesity: BMI >23 kg/m^2^. BMI, body mass index.

Six mutually exclusive malnutrition burden categories were derived using the combinations of the three BMI statuses and two anaemia statuses. These are: (i) no burden, including women with normal weight and no anaemia (‘no malnutrition burden’); (ii) single burdens, including normal weight with anaemia (‘anaemia only’), underweight without anaemia (‘underweight only’) and overweight/obesity without anaemia (‘overweight/obesity only’); and iii) double burdens, including underweight with anaemia (‘underweight-anaemia double burden’) and overweight/obesity with anaemia (‘overweight/obesity-anaemia double burden’) (see figure 1).

When presenting prevalence estimates, the terms ‘anaemia overall’, ‘underweight overall’ and ‘overweight/obesity’ overall are also used. These are not mutually exclusive categories. For example, any woman with underweight, regardless of anaemia status, is included in ‘overall underweight’ estimates.

### Conceptual framework for the selection of potential risk factors

To guide variable selection, a systematic search of the literature on risk factors for the co-occurrence of anaemia with underweight or overweight/obesity among reproductive-age women in South Asia was conducted on 17 May 2024. Details on the search are provided in the Supplementary Materials. Among papers identified for the overweight/obesity-anaemia double burden, only urbanicity and increasing age, wealth and education were consistently found to be risk factors. No corroborated risk factors for the underweight-anaemia double burden were identified.

To supplement these findings, risk factors for each of underweight, overweight/obesity and anaemia were extracted from six international frameworks and synthesised to provide a list of factors with theoretical importance for occurrence of anaemia, underweight or overweight/obesity. These factors were then mapped to variables available in the NFHS-5 (online supplemental Table 2).

**Figure 2:**
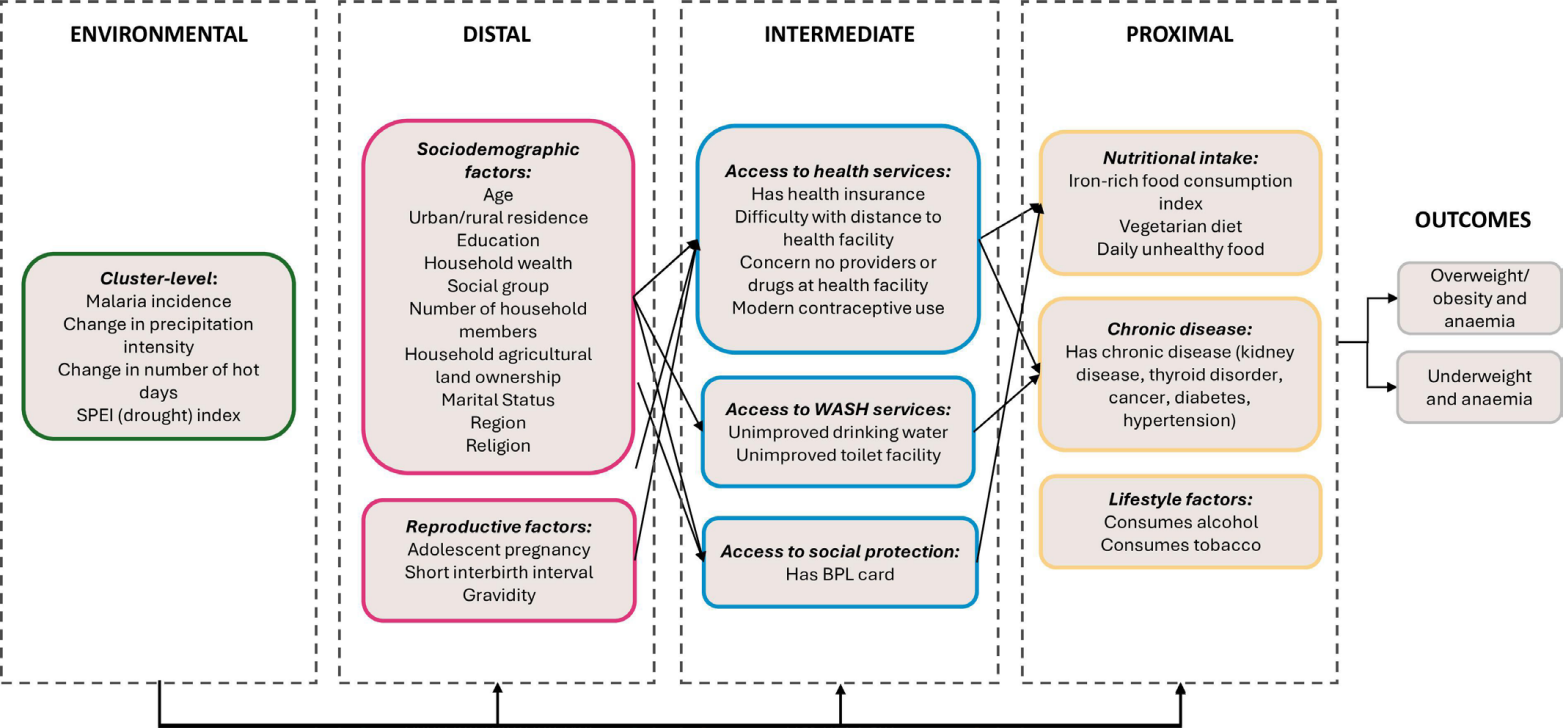
Conceptual framework identifying potential risk factors for the double burdens of anaemia with underweight or of anaemia with overweight/obesity from variables available in the NFHS-5, 2019–2021. Notes: WASH=water, sanitation and hygiene. BPL card=below poverty line card which entitles family to subsidised food grain. Potential risk factors were selected by synthesising determinants of anaemia, underweight or overweight/obesity identified in six international frameworks: UNICEF 2020 framework for Maternal and Child Nutrition,^45^ UNICEF 2013 framework for Maternal and Child Undernutrition,^67^ the Foresight 2007 Obesity System Map,^68^ the ROOTS obesity framework,^69^ Balarajan *et al*^34^ and WHO accelerating anaemia reduction.^14^ NFHS, National Family and Health Survey.

The conceptual framework developed informed variable selection and is presented in figure 2. The framework identified 30 DHS variables available for all women of reproductive age as potential risk factors for the double burdens. The risk factors were organised across categories of proximal (nutritional intake, chronic disease and lifestyle), intermediate (access to health, WASH and social protection services), distal (sociodemographic and reproductive) and environmental (cluster level) factors.^45^

It is important to note that appropriate proxies for many key determinants of malnutrition identified in the six international frameworks were not available in the DHS. These are listed in online supplemental Table 2) but are not included in the conceptual framework. In particular, we note a lack of data on haemoglobinopathies, inflammation and non-malarial infections, which are known to be important non-nutritional drivers of anaemia. Similarly, physical activity and quantity of food consumption data are important contributors to the energy balance underlying underweight and overweight/obesity but are not present in the DHS. Nonetheless, the breadth of variables that are available in the DHS and are included in our conceptual framework remains a strength of this dataset.

The term ‘risk factors’ is used throughout this study. As a cross-sectional analysis, the term does not imply definitive causality in this context, but is used in its conventional epidemiological sense to refer to variables that are associated with the outcomes and have biological or social plausibility.

### Individual-level variables

For the purposes of this study, iron-rich food intake was specified as the sum of intake of meat, fish, beans/pulses, eggs and leafy green vegetables, weighted by frequency of consumption (1=weekly, 2=daily). A one-unit increase in iron-rich food index corresponds to eating from one additional food group weekly or changing from weekly to daily consumption of one food group. A vegetarian diet is defined as lacto-vegetarian, including women who report to never consume meat, fish or eggs. Unhealthy food consumption is specified as women who consume either fried foods or aerated drinks daily. Alcohol consumption is specified as ever consumption of alcohol. Tobacco consumption is specified as consuming tobacco in any form. While specified in the contextual framework, chronic disease variables were not included in the model as all have evidenced bidirectional relationships with underweight or overweight/obesity.

Having health insurance is specified as being covered by any government or private insurance. Concern with distance or no providers/drugs at the health facility is based on self-report of these as being ‘a big problem’. Modern contraceptive use, unimproved toilet facility and unimproved drinking water source were categorised according to DHS recommendations.^46^ BPL (‘below poverty line’) card possession is based on household self-report and entitles the household to subsidised food rations.

Age is categorised in 5-year intervals. Residence corresponds to DHS categorisation of survey cluster as urban or rural. Education is categorised according to highest grade of school completed. Wealth quintile is determined according to standard DHS methodology based on household items, assets and construction materials. Social group is specified according to government classification of disadvantaged caste or tribe or other backward class (OBC) membership. Number of household members and household ownership of agricultural land is based on household head self-report. Marital status is based on individual self-report. The region is categorised according to the state’s membership of India’s six zonal councils. Religion is specified as Hindu, Muslim or other. Adolescent birth is specified for women who gave birth before the age of 20. A short birth interval is specified for women for whom the interval between any past births was shorter than 24 months. Gravidity is specified as number of children who were ever born to the woman. Online supplemental table 4 provides a list of the derivation of all variables analysed.

### Cluster-level variables

The international anaemia, overweight/obesity and underweight frameworks commonly included climate as a determinant of malnutrition. The World Bank identifies heat waves, droughts, cyclones and other causes of flooding as the major climate risks facing India.^47^ This study proxies these climate risks using historical geospatial data from the World Bank’s Climate Change Knowledge Portal.^48^ Heat waves are proxied through the average number of days per year in which the temperature was over 40°C (30°C in Himalayan region), approximating the India Meteorological Department’s (IMD) heatwave definition.^49^ Flooding is proxied by average precipitation (millimetres) in the 5% wettest days annually. Changes in each of the average annual number of hot days and average annual precipitation intensity, between a baseline of 1950–2000 and the recent 2010–2020 period, are calculated to form environmental anomaly variables. Drought risk is proxied using the 2010–2020 average of the Standardised Precipitation Evapotranspiration Index (SPEI) value, a measure of the integrated water deficit in a location compared with a long-term average.

Malaria infection risk is proxied using cluster-level data from the DHS geospatial covariates dataset and DHS survey clusters are classified according to the WHO thresholds for cases per 1000 inhabitants.^50^

These four variables are grouped as ‘cluster-level’ variables as they are applied to survey clusters as opposed to individuals and are included in the multilevel model using level two weights. Full derivation is presented in online supplemental Table 4).

### Statistical analysis

Using data from all survey years, the prevalences of anaemia, underweight, overweight/obesity and the double burdens were calculated for each district (NFHS-4 and NFHS-5) or state (NFHS-3) and mapped (NFHS-3 was not designed to be representative at the district level).

The primary model for risk factor analysis presented in this paper used data from only the final survey round (NFHS-5, 2019–2021). Unweighted counts and weighted percentages (for categorical variables) and medians and interquartile ranges (for non-normal continuous variables) were calculated for all potential risk factors across the six outcomes. Significant variation in the distribution of risk factors across levels of the outcome variable was assessed using a χ2 test with Rao and Scott’s second-order correction (categorical variables) or a Wilcoxon rank-sum test for complex survey samples (continuous variables).

Linearity of the association of continuous variables (iron-rich food index, gravidity) with the log of all outcomes was assessed using orthogonal polynomials across quintiles of continuous variables. Variables for which an adjusted F-test was not significant at the 5% level for all outcomes were categorised.

Correlation between potential risk factors was assessed by estimating standardised covariances as recommended for complex survey designs.^51^ Risk factors showing high collinearity (>0.6) were assessed and one of the pair removed after considering theoretical relevance. Multicollinearity was assessed and variables with a variance inflation factor (VIF) larger than five were further examined.

Univariable multinomial logistic regression was performed to assess the association of each potential risk factor with the single and double burden outcomes versus those with no malnutrition burden. Risk factors for which an adjusted Wald test showed statistical significance at the 5% level were retained for multivariable modelling.

In the primary risk factor analysis, a multilevel logistic regression model was used to assess the associations of all potential risk factors with the outcomes in the NFHS-5 (2019–2021) survey. To account for the constancy of environmental variables within clusters, the regression was modelled with a latent variable at the cluster level, and women were nested within clusters (multilevel model). Five single and double burden binary malnutrition outcomes with a common baseline outcome (no malnutrition burden) were modelled simultaneously using logistic regression. This configuration closely approximates a multinomial model and was adopted as this requires less computational power. Joint adjusted Wald tests were used to assess the statistical significance of potential risk factors.

Models were sequentially adjusted for the four sets of variables presented in the conceptual framework (figure 2) from proximal, intermediate and distal to environmental factors. Receiver operating curves for each sequential adjustment were plotted and the area under the Curve statistic interpreted for contribution of each sequential adjustment to model discrimination.

A complete case analysis was conducted as overall missingness was low (1.1%), and percentage missing did not vary substantially by outcome group for any variable (table 1).

In a secondary analysis, a multiple group analysis was run in the NFHS-5 (2019–2021), NFHS-4 (2015–2016) and NFHS-3 (2005–2006) rounds to assess changes in risk factors over time. These models were stratified by survey round and included only variables available in all three rounds, so did not include cluster-level environmental variables or unhealthy food consumption (NFHS-3 survey responses are not linked to GPS locations restricting the ability to link to externally available environmental variables). Differences between ORs for determinants across the three models for different survey rounds were assessed using a Wald OR test.

All analyses were adjusted for survey design and clustering using Stata’s svyset command, with separate weighting for level 1 (individual) and level 2 (cluster) variables in the multilevel model. Level weighting derivations are presented in the Supplementary Materials. Wald tests and F tests were used throughout as appropriate for complex survey designs. Taylor linearised standard errors were used.

All p values in the multivariable regressions were interpreted at a conservative Bonferroni-corrected 5% alpha threshold to account for multiple testing in this exploratory analysis, corresponding to a threshold of 0.00179 in the primary risk factor analysis (threshold=0.05/28, given 28 potential risk factors used in analysis). Clinical significance over a 5% effect size threshold was also used to inform interpretation of coefficients to avoid over-interpretation of potentially chance findings.

In response to current uncertainty regarding the health impacts of mild anaemia only,^42^ a sensitivity analysis for the primary risk factor model was re-run with anaemia defined using a lower haemoglobin threshold. Anaemia was defined only as moderate/severe anaemia (haemoglobin <11.0 g/dL), and those with mild or no anaemia (haemoglobin ≥11.0 g/dL) were defined as not having anaemia.

Analysis was conducted in Stata 18.0^52^ with plotting in R 4.4.0.^53^

## Results

### Spatial distribution of the double burdens of malnutrition

**Figure 3:**
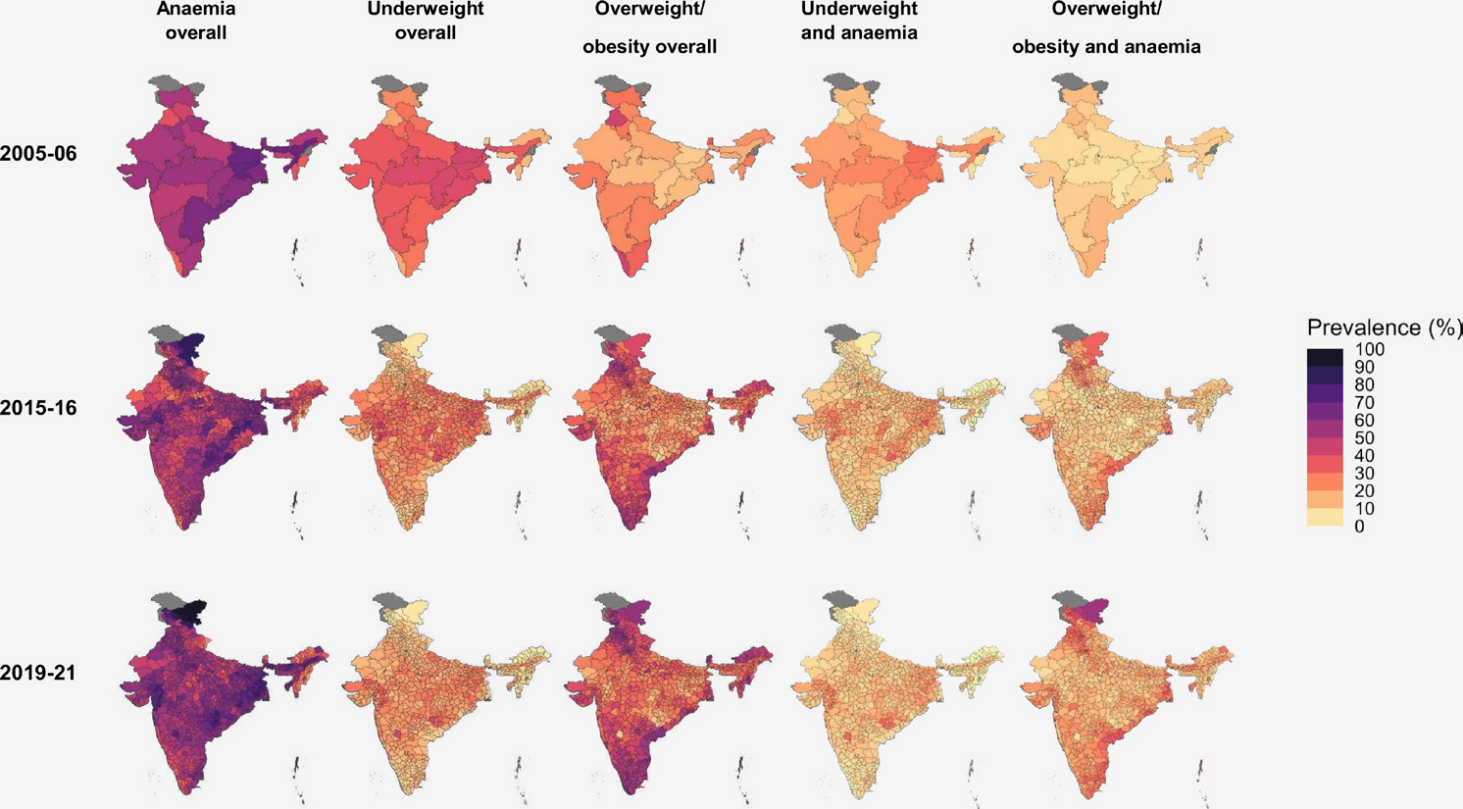
Prevalence of the double burdens of malnutrition and the overall burdens of anaemia, underweight and overweight/obesity among women of reproductive age by district (2019–2021, NFHS-5; 2015–2016, NFHS-4) or state (2005–2006, NFHS-3) of India. Notes: NFHS 2005–2006 was only sampled to be representative at the state level, while 2015–2016 and 2019–2021 were sampled to be representative at the district level. Estimates are presented at these respective levels. Nagaland is excluded from prevalence estimates in 2005–2006 as anaemia measurements were not available. Unlike elsewhere in the paper, anaemia, underweight and overweight/obesity burdens are presented as ‘overall’, meaning total estimates of prevalence for these burdens are presented irrespective of anaemia status. Anaemia: haemoglobin concentration <12.0 g/dL. Underweight: BMI <18.5 kg/m^2^. Overweight/obesity: BMI >23 kg/m^2^. Pregnant and up to 3 months post-partum women are excluded from estimates. BMI, body mass index; DHS, Demographic and Health Surveys; NFHS, National Family and Health Survey.

Figure 3 displays the state-level or district-level prevalence of each of the double burdens alongside the overall prevalence of anaemia, underweight and overweight/obesity in each survey round (2005–2006, 2015–2016, 2019–2021).

The underweight-anaemia double burden was most prevalent across Western, Central, Eastern and lowland North Eastern regions of India, with hotspots in Gujarat, Chhattisgarh and Jharkhand where prevalence in some districts exceeded 25% in both 2015–2016 and 2019–2021. The state with the highest prevalence in 2005–2006 was Bihar (Eastern India), with 29% prevalence.

The overweight/obesity-anaemia double burden was most prevalent in Southern, Northern and coastal Eastern regions. Highest prevalence was found in districts in Ladakh and Jammu and Kashmir (Northern India) with prevalence of up to 55% in 2019–2021. The districts with the highest prevalence in 2015–2016 were found in Punjab (Northern India), with prevalence up to 43% in each district. In 2005–2006, the states with the highest prevalences were Sikkim (North Eastern India) and Tamil Nadu (Southern India), where prevalence still remained under 19%.

Table 2 shows that the prevalence of overall underweight almost halved between 2005–2006 and 2019–2021 (34% vs 18%). In the same period, overall overweight/obesity became 75% more prevalent, and prevalence exceeded 20% in all states in 2019–2021. Overall anaemia prevalence did not decline nationally (55% in 2005–2006, 53% in 2015–2016 and 57% in 2019–2021) and has become substantially more prevalent in some districts. Due to the high and less variable prevalence of anaemia, the distribution of both the underweight-anaemia and overweight/obesity-anaemia double burdens was largely reflections of the spatial distributions of BMI in the country, being most prevalent in areas in which underweight or overweight/obesity were most prevalent, respectively.

**Table 1:** Sample characteristics of women of reproductive age across all malnutrition outcomes, NFHS-5, 2019–2021

		No burden of malnutrition		Single burdens of malnutrition			Double burdens of malnutrition	
	Overall	Normal weight only	Anaemia only	Underweight only	Overweight/obesity only	Underweight- anaemia	Overweight/obesity-anaemia	p value*
N (%)	632 591	114 674 (17)	175 516 (27)	37 966 (6.3)	107 178 (17)	70 313 (11)	126 944 (21)	
Proximal factors (individual level)								
Iron-rich food index, median (IQR)	4.00 (3.00, 5.00)	4.00 (3.00, 5.00)	4.00 (3.00, 5.00	4.00 (3.00, 5.00)	4.00 (3.00, 5.00)	4.00 (3.00, 5.00)	4.00 (3.00, 6.00)	<0.001
Vegetarian	165 408 (27)	30 386 (28)	43 139 (26)	10 785 (28)	29 022 (27)	18 634 (26)	33 442 (26)	<0.001
Consumes unhealthy food or beverages daily	72 651 (9.4)	13 325 (8.9)	20 958 (10.0)	3469 (8.4)	12 645 (9.1)	6919 (8.8)	15 335 (10)	<0.001
Drinks alcohol	12 326 (0.8)	2074 (0.7)	3774 (1.0)	444 (0.6)	2300 (0.6)	1428 (1.1)	2306 (0.7)	<0.001
Uses tobacco	41 750 (4.2)	7412 (3.7)	12 569 (4.8)	2011 (4.0)	6720 (3.2)	5574 (6.0)	7464 (3.8)	<0.001
Intermediate factors (individual level)								
Covered by health insurance	207 850 (31)	37 976 (31)	59 796 (31)	12 148 (29)	34 303 (31)	23 750 (30)	39 877 (31)	<0.001
Distance to health facility is a big concern	166 905 (23)	30 125 (23)	50 844 (26)	10 511 (25)	23 689 (19)	21 409 (28)	30 327 (21)	<0.001
No providers or drugs at health facility is a big concern	307 874 (46)	55 424 (45)	90 805 (49)	18 986 (47)	46 351 (40)	38 025 (51)	58 283 (43)	<0.001
Modern contraception user	272 680 (45)	45 973 (42)	69 835 (43)	11 310 (30)	55 919 (55)	22 513 (33)	67 130 (56)	<0.001
Missing	8 (<0.1)	2 (<0.1)	2 (<0.1)	3 (<0.1)	0 (0)	1 (<0.1)	0 (0)	
Unimproved drinking water source	39 153 (4.1)	7675 (4.2)	13 150 (4.9)	2264 (4.5)	4960 (3.0)	5011 (5.2)	6093 (3.1)	<0.001
Unimproved toilet facility	124 959 (21)	23 117 (22)	40 330 (24)	9771 (26)	13 585 (14)	21 082 (30)	17 074 (14)	<0.001
Missing	1 (<0.1)	0 (0)	0 (0)	0 (0)	1 (<0.1)	0 (0)	0 (0)	
Household has BPL card	313 088 (46)	56 865 (46)	93 150 (49)	20 005 (50)	46 103 (40)	39 473 (53)	57 492 (43)	<0.001
Distal factors (individual level)								
Age in 5-year groups								<0.001
15–19	109 156 (17)	23 307 (21)	37 319 (21)	12 780 (34)	6404 (5.8)	21 410 (31)	7936 (6.1)	
20–24	92 059 (14)	19 733 (17)	29 392 (17)	8460 (22)	9212 (8.8)	14 390 (21)	10 872 (8.5)	
25–29	96 035 (15)	18 816 (16)	28 026 (16)	5363 (14)	15 232 (14)	10 524 (15)	18 074 (14)	
30–34	89 549 (14)	15 273 (13)	23 050 (13)	3694 (9.5)	18 504 (17)	7438 (10)	21 590 (17)	
35–39	90 646 (14)	14 547 (13)	22 098 (13)	2971 (7.6)	20 266 (19)	6528 (9.0)	24 236 (19)	
40–44	76 308 (12)	11 244 (9.9)	17 707 (10)	2363 (6.0)	18 164 (17)	4905 (6.7)	21 925 (17)	
45–49	78 838 (13)	11 754 (10)	17 924 (10)	2335 (6.0)	19 396 (18)	5118 (7.2)	22 311 (18)	
Residence								<0.001
Urban	156 079 (32)	26 982 (30)	34 511 (27)	7273 (24)	36 403 (42)	11 628 (22)	39 282 (40)	
Rural	476 512 (68)	87 692 (70)	141 005 (73)	30 693 (76)	70 775 (58)	58 685 (78)	87 662 (60)	
Education								<0.001
No education	149 914 (23)	26 205 (23)	44 577 (25)	7962 (20)	24 022 (22)	17 601 (24)	29 547 (22)	
Incomplete primary	75 266 (12)	13 233 (11)	20 970 (12)	3694 (9.5)	13 398 (13)	7894 (11)	16 077 (13	
Incomplete secondary	299 892 (47)	53 812 (45)	82 810 (46)	19 045 (49)	50 009 (45)	34 028 (48)	60 188 (47)	
Complete secondary or higher	107 519 (18)	21 424 (20)	27 159 (16)	7265 (21)	19 749 (20)	10 790 (17)	21 132 (18)	
Wealth index quintile								<0.001
Poorest	120 672 (19)	24 683 (19)	45 808 (24)	9894 (24)	11 708 (9.2)	22 602 (29)	15 471 (11)	
Poorer	132 705 (20)	27 310 (21)	43 577 (23)	9719 (24)	18 900 (15)	18 136 (25)	23 275 (16)	
Middle	130 026 (21)	24 182 (21)	36 728 (21)	8000 (21)	23 110 (21)	13 718 (20)	27 942 (21)	
Richer	125 957 (21)	21 190 (20)	29 060 (18)	6271 (18)	25 731 (26)	9951 (16)	30 374 (25)	
Richest	123 231 (20)	17 309 (18)	20 343 (14)	4082 (12)	27 729 (30)	5906 (10)	29 882 (26)	
Social group								
Schedule caste	121 453 (22)	21 512 (22)	34 102 (23)	8358 (23)	18 482 (19)	15 239 (24)	23 760 (21)	
Schedule tribe	120 211 (9.5)	23 068 (8.7)	40 427 (13)	5734 (9.5)	16 723 (5.3)	15 505 (15)	18 754 (6.7)	
OBC	240 945 (43)	45 163 (45)	62 992 (40)	16 748 (46)	42 348 (46)	27 350 (41)	46 344 (41)	
Other caste, no caste or unsure	149 982 (26)	24 931 (24)	37 995 (24)	7126 (21)	29 625 (30)	12 219 (20)	38 086 (31)	
Number of household members								<0.001
3 or fewer	109 806 (18)	19 218 (16)	28 494 (16)	5193 (14)	21 938 (20)	9962 (14)	25 001 (21)	
4 or 5	284 889 (45)	50 744 (44)	78 878 (44)	16 318 (43)	49 389 (46)	30 548 (43)	59 012 (46)	
6 or more	237 896 (38)	44 712 (40)	68 144 (39)	16 455 (44)	35 851 (34)	29 803 (42)	42 931 (33)	
Owns land usable for agriculture	303 863 (42)	56 109 (43)	89 459 (44)	19 317 (47)	45 656 (36)	36 453 (46)	56 869 (37)	<0.001
Current marital status								<0.001
Never in union	167 582 (25)	36 776 (31)	54 715 (29)	17 665 (47)	13 925 (12)	28 355 (41)	16 146 (11)	
Married	436 823 (71)	73 450 (65)	113 109 (66)	19 157 (50)	87 743 (83)	39 367 (56)	103 997 (83)	
Divorced/separated/widowed	28 186 (4.5)	4448 (3.8)	7692 (4.5)	1144 (2.8)	5510 (5.1)	2591 (3.6)	6801 (5.6)	
Region								<0.001
Northern	116 377 (13)	21 059 (15)	34 678 (14)	6031 (12)	22 983 (15)	10 567 (11)	32 415 (15)	
North Eastern	92 837 (3.8)	20 431 (4.1)	28 279 (4.8)	3285 (2.7)	17 130 (3.0)	6612 (3.5)	17 100 (3.5)	
Central	156 053 (25)	31 051 (30)	40 189 (24)	11 624 (31)	22 589 (24)	17 637 (24)	21 607 (18)	
Eastern	102 651 (23)	15 830 (20)	33 867 (28)	6608 (21)	11 345 (16)	16 072 (28)	18 929 (23)	
Western	64 571 (14)	10 569 (13)	17 261 (14)	4737 (16)	9877 (13)	10 010 (18)	12 117 (14)	
Southern	100 102 (21)	15 734 (18)	21 242 (16)	5681 (18)	23 254 (29)	9415 (16)	24 776 (26)	
Religion								<0.001
Hindu	478 556 (82)	85 123 (82)	133 275 (83)	31 406 (85)	77 304 (79)	58 654 (85)	92 794 (80)	
Muslim	76 718 (13)	13 167 (13)	21 466 (13)	3863 (12)	13 332 (14)	6584 (11)	18 306 (14)	
Other	77 317 (5.1)	16 384 (4.7)	20 775 (4.6)	2697 (3.5)	16 542 (6.4)	5075 (4.1)	15 844 (6.1)	
Adolescent birth (<20 years)	179 776 (30)	29 955 (27)	48 617 (30)	8281 (22)	33 984 (34)	17 455 (25)	41 484 (36)	<0.001
Short birth interval (<24 months)	162 044 (26)	27 055 (24)	41 842 (24)	6983 (18)	32 987 (31)	14 390 (20)	38 787 (31)	<0.001
Gravidity, median (IQR)	2 (0, 3)	2 (0, 3)	2 (0, 3)	0 (0, 2)	1 (0, 2)	2 (1, 3)	2 (1, 3)	<0.001
Environmental factors (cluster level)								
Change in annual number of hot days								<0.001
Little change (<5 days more than average)	297 811 (50)	49 963 (47)	81 305 (49)	15 289 (43)	53 928 (55)	29 313 (44)	68 013 (56)	
More hot days (5 to 10 days more than average)	180 670 (31)	33 938 (33)	51 332 (32)	13 427 (37)	26 585 (27)	24 497 (36)	30 891 (27)	
Many more hot days (>10 days more than average)	150 187 (19)	29 942 (20)	42 102 (19)	9054 (20)	25 539 (18)	16 248 (20)	27 302 (17)	
Missing	3923 (0.3)	831 (0.3)	777 (0.3)	196 (0.3)	1126 (0.4)	255 (0.3)	738 (0.4)	
Change in annual precipitation intensity								<0.001
Much less precipitation (< -200mm less than average)	24 766 (0.9)	5223 (0.8)	6770 (1.0)	814 (0.6)	5403 (0.8)	1498 (0.8)	5058 (0.9)	
Less precipitation (-100mm to -200mm less than average)	64 690 (6.9)	11 917 (7.4)	18 683 (7.3)	3205 (6.9)	11 133 (6.6)	5639 (6.3)	14 113 (6.8)	
Little change (-100mm to +100mm change)	458 155 (79)	84 338 (80)	126 782 (79)	28 776 (80)	76 427 (78)	51 019 (78)	90 813 (79)	
More precipitation (> 100mm more than average)	82 990 (13)	12 863 (12)	22 772 (13)	5051 (13)	13 819 (15)	11 971 (15)	16 514 (14)	
Missing	1990 (0.3)	333 (0.3)	509 (0.2)	120 (0.3)	396 (0.3)	318 696 (0.2)	446 (0.3)	
SPEI (drought) index								<0.001
First tertile (little change)	178 062 (29)	39 040 (32)	62 172 (30)	13 490 (33)	30 999 (26)	24 512 (30)	39 981 (24)	
Second tertile (drier than average)	224 293 (35)	40 673 (32)	60 906 (32)	12 985 (32)	33 530 (28)	24 080 (33)	38 031 (28)	
Third tertile (drier than average)	226 313 (36)	34 628 (36)	51 929 (38)	11 371 (35)	42 253 (46)	21 535 (37)	48 486 (48)	
Missing	1990 (0.3)	333 (0.3)	509 (0.2)	120 (0.3)	396 (0.3)	186 (0.2)	446 (0.3)	
Malaria incidence								<0.001
Low transmission	455 182 (78)	82 100 (79)	121 437 (77)	27 202 (79)	81 228 (83)	46 916 (75)	96 299 (81)	
Moderate transmission	92 991 (13)	17 514 (13)	26 858 (14)	5775 (13)	14 850 (12)	11 376 (15)	16 618 (12)	
High transmission	78 428 (7.9)	13 786 (7.5)	26 009 (9.4)	4706 (8.2)	9537 (5.9)	11 603 (11)	12 787 (6.9)	
Missing	5990 (0.9)	1274 (1.0)	1212 (0.7)	283 (0.7)	418 (1.2)	1563 (0.5)	1240 (1.0)	

* χ2 test with Rao and Scott’s second-order correction; Wilcoxon rank-sum test for complex survey samples.

BPL, below poverty line; NFHS, National Family and Health Survey; SPEI, Standardised Precipitation Evapotranspiration Index.

**Table 2:** Prevalence and 95% CIs of malnutrition over time among women of reproductive age in India, 2005–2006, 2015–2016 and 2019–2021, NFHS

	Single burdens			Double burdens	
	Prevalence (95% CI)			Prevalence (95% CI)	
Year	Anaemia only	Underweight only	Overweight/obesity only	Underweight-anaemia	Overweight/obesity-anaemia
2005–2006	24.7 (24.3–25.1)	13.4 (13.0–13.7)	11.8 (11.5–12.2)	20.8 (20.3–21.3)	9.7 (9.4–10.0)
2015–2016	25.0 (24.8–25.1)	8.6 (8.5–8.7)	17.1 (16.9–17.3)	13.1 (13.0–13.3)	16.6 (16.5–16.8)
2019–2021	26.6 (26.4–26.8)	6.3 (6.2–6.4)	17.4 (17.2–17.6)	11.4 (11.3–11.5)	20.9 (20.7–21.1)

Anaemia: haemoglobin concentration <12.0 g/dL. Underweight: BMI <18.5 kg/m2. Overweight/obesity: BMI >23kg/m2. BMI: Body Mass Index. Pregnant and up to 3 months post-partum women are excluded from estimates. All single and double burden categories are mutually exclusive. ‘Overall’ anaemia, underweight and overweight/obesity prevalence can be calculated by summing the appropriate single and double burden categories.

Data sources: NFHS-3 (2005–2006, n = 104 609), NFHS-4 (2015–2016, n = 637 610), NFHS-5 (2019–2021, n = 646 210).

NFHS, National Family and Health Survey.

### Sample characteristics

**Figure 4:**
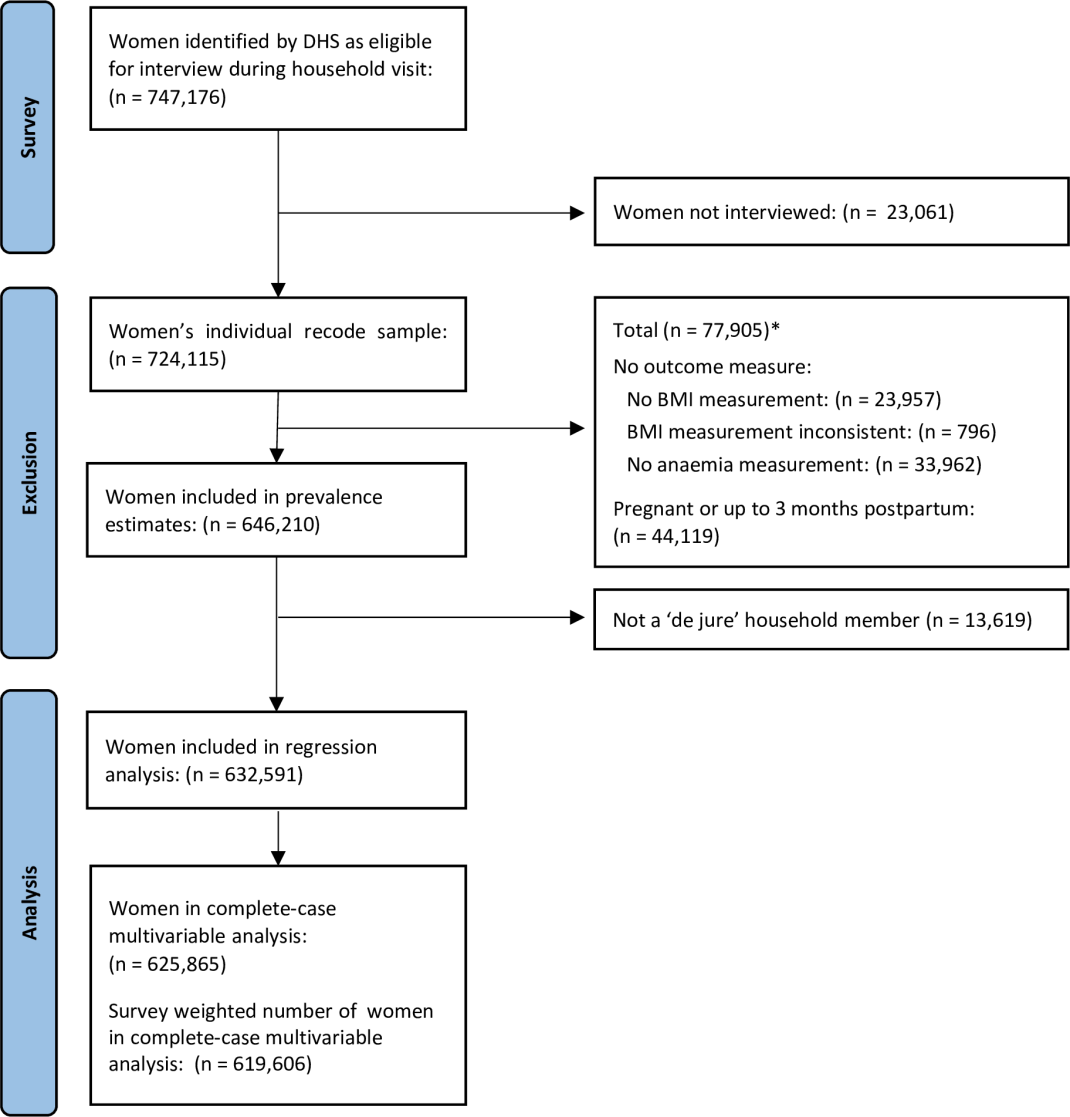
Flow diagram of the selection of the survey and study participants from the NFHS-5, 2019–2021. *Notes: Numbers presented may not sum to the total as some individuals met more than one of the exclusion criteria presented. AUC, area under the curve.

For the primary risk factor analysis, 632 591 women met the final inclusion criteria. Of these, 625 865 had complete data across all selected risk factors and were included in multilevel multivariable modelling using complete case analysis (see figure 4). Inclusion flow diagrams for the secondary analyses using the NFHS-5, NFHS-4 and NFHS-3 datasets are presented in online supplemental figures 1-3.

Characteristics of the NFHS-5 study population are presented in table 1. Only 17% of reproductive-age women in India did not have any of the forms of malnutrition investigated. In 2019–2021, 21% had the double burden of overweight/obesity-anaemia, and 11% had the double burden of underweight-anaemia. Compared with individuals with no burden of malnutrition (normal weight and not anaemic), overweight/obesity-anaemia was more prevalent among older, richer and urban women with reproductive risk factors (adolescent birth or short birth interval), while underweight-anaemia was more prevalent among younger, poorer, rural and unmarried women with worse access to WASH services.

### Primary analysis: risk factors for the single and double burdens of malnutrition in 2019–2021

Gravidity and iron-rich food consumption index were linearly associated with all outcomes, while non-linear associations were found with malaria incidence, SPEI (drought) index and change in number of hot days and precipitation intensity, which were therefore categorised. Age and number of household members were linearly associated with outcomes but are presented in categorical form for ease of interpretation.

Gravidity was removed due to its high collinearity with age (r=0.65) and short birth intervals (r=0.62) and moderate collinearity (r>=0.45) with marital status, education and adolescent birth.

In univariable analyses, all potential risk factors were significantly associated with at least one of the outcomes and were retained for multivariable modelling. There was no evidence of multicollinearity in the final multivariable model (VIF <2.3).

Table 3 presents the adjusted ORs for the associations of each potential risk factor with the single and double malnutrition burdens versus no malnutrition burden. Risk factors for the double burdens in 2019–2021.

**Table 3:** Associations of potential risk factors with the single and double burdens of malnutrition compared to no malnutrition burden, NFHS-5, 2019–2021

	Single burdens OR (95% CI)			Double burdens OR (95% CI)	
	Anaemia only	Underweight only	Overweight/obesity only	Underweight-anaemia	Overweight/obesity-anaemia
N =	175 516	37 966	107 178	70 313	126 944
Proximal factors (individual level)					
Iron-rich food consumption index			***	***	
	0.99 (0.98, 1.00)	0.98 (0.97, 0.99)	1.02 (1.01, 1.03)	0.98 (0.97, 0.99)	1.02 (1.01, 1.03)
Vegetarian				***	***
No	1.00 (ref)	1.00 (ref)	1.00 (ref)	1.00 (ref)	1.00 (ref)
Yes	1.04 (1.00, 1.07)	1.00 (0.95, 1.04)	0.99 (0.96, 1.03)	1.07 (1.03, 1.11)	1.08 (1.04, 1.13)
Daily unhealthy food consumption					
No	1.00 (ref)	1.00 (ref)	1.00 (ref)	1.00 (ref)	1.00 (ref)
Yes	1.06 (1.01, 1.11)	1.04 (0.98, 1.12)	1.05 (1.00, 1.11)	1.02 (0.96, 1.08)	1.07 (1.01, 1.13)
Drinks alcohol					
No	1.00 (ref)	1.00 (ref)	1.00 (ref)	1.00 (ref)	1.00 (ref)
Yes	0.92 (0.83, 1.03)	0.93 (0.78, 1.10)	0.96 (0.84, 1.10)	0.99 (0.87, 1.12)	0.86 (0.75, 0.99)
Uses tobacco	***	***	***	***	
No	1.00 (ref)	1.00 (ref)	1.00 (ref)	1.00 (ref)	1.00 (ref)
Yes	1.11 (1.05, 1.17)	1.39 (1.27, 1.51)	0.88 (0.83, 0.94)	1.63 (1.51, 1.75)	0.95 (0.89, 1.01)
Intermediate factors (individual level)					
Has health insurance			***		***
No	1.00 (ref)	1.00 (ref)	1.00 (ref)	1.00 (ref)	1.00 (ref)
Yes	1.04 (1.01, 1.07)	1.03 (0.99, 1.07)	0.93 (0.90, 0.96)	1.02 (0.98, 1.06)	0.92 (0.89, 0.96)
Difficulty with distance to health facility					
Not a big concern	1.00 (ref)	1.00 (ref)	1.00 (ref)	1.00 (ref)	1.00 (ref)
Big concern	1.04 (1.01, 1.07)	1.04 (1.00, 1.09)	0.98 (0.94, 1.01)	1.06 (1.01, 1.10)	1.01 (0.98, 1.05)
Concern no providers or drugs at health facility				***	
Not a big concern	1.00 (ref)	1.00 (ref)	1.00 (ref)	1.00 (ref)	1.00 (ref)
Big concern	1.03 (1.00, 1.06)	1.01 (0.97, 1.05)	0.98 (0.95, 1.01)	1.06 (1.03, 1.10)	1.02 (0.99, 1.06)
Modern contraceptive use		***	***	***	
No	1.00 (ref)	1.00 (ref)	1.00 (ref)	1.00 (ref)	1.00 (ref)
Yes	1.02 (0.99, 1.05)	0.92 (0.88, 0.96)	0.91 (0.88, 0.94)	0.87 (0.83, 0.90)	0.96 (0.93, 0.99)
Unimproved drinking water					
No	1.00 (ref)	1.00 (ref)	1.00 (ref)	1.00 (ref)	1.00 (ref)
Yes (unimproved)	1.06 (1.00, 1.12)	1.01 (0.93, 1.09)	0.99 (0.92, 1.06)	1.04 (0.97, 1.12)	0.96 (0.90, 1.04)
Unimproved toilet facility			***		***
No	1.00 (ref)	1.00 (ref)	1.00 (ref)	1.00 (ref)	1.00 (ref)
Yes (unimproved)	1.00 (0.96, 1.03)	0.99 (0.95, 1.04)	0.93 (0.89, 0.97)	1.06 (1.02, 1.10)	0.90 (0.86, 0.95)
Has BPL card			***		***
No	1.00 (ref)	1.00 (ref)	1.00 (ref)	1.00 (ref)	1.00 (ref)
Yes	1.00 (0.98, 1.03)	0.99 (0.96, 1.03)	0.87 (0.84, 0.90)	1.04 (1.01, 1.08)	0.93 (0.90, 0.97)
Distal factors (individual level)					
Age	***	***	***	***	***
15-19	1.12 (1.06, 1.19)	1.56 (1.45, 1.68)	0.44 (0.41, 0.47)	1.63 (1.53, 1.75)	0.50 (0.47, 0.54)
20–24	1.02 (0.98, 1.07)	1.37 (1.29, 1.47)	0.62 (0.59, 0.66)	1.33 (1.25, 1.40)	0.63 (0.60, 0.67)
25–29	1.00 (ref)	1.00 (ref)	1.00 (ref)	1.00 (ref)	1.00 (ref)
30–35	1.00 (0.96, 1.05)	0.82 (0.76, 0.87)	1.56 (1.48, 1.63)	0.78 (0.74, 0.83)	1.52 (1.45, 1.60)
35–39	0.98 (0.94, 1.03)	0.66 (0.61, 0.71)	1.83 (1.74, 1.93)	0.69 (0.65, 0.74)	1.85 (1.76, 1.95)
40–45	1.02 (0.97, 1.08)	0.66 (0.61, 0.71)	2.14 (2.03, 2.26)	0.63 (0.58, 0.67)	2.23 (2.11, 2.35)
45–49	1.00 (0.95, 1.05)	0.62 (0.57, 0.67)	2.29 (2.17, 2.41)	0.64 (0.60, 0.69)	2.33 (2.21, 2.46)
Residence		***	***		***
Urban residence	1.00 (ref)	1.00 (ref)	1.00 (ref)	1.00 (ref)	1.00 (ref)
Rural residence	0.97 (0.93, 1.02)	1.11 (1.05, 1.18)	0.91 (0.87, 0.96)	1.06 (1.00, 1.13)	0.89 (0.84, 0.94)
Education	***	***	***	***	***
No education	1.00 (ref)	1.00 (ref)	1.00 (ref)	1.00 (ref)	1.00 (ref)
Incomplete primary	0.98 (0.94, 1.02)	0.83 (0.78, 0.88)	1.15 (1.10, 1.21)	0.85 (0.80, 0.89)	1.21 (1.15, 1.27)
Incomplete secondary	0.98 (0.94, 1.01)	0.81 (0.77, 0.86)	1.30 (1.24, 1.35)	0.78 (0.75, 0.82)	1.37 (1.32, 1.43)
Complete secondary and higher	0.86 (0.82, 0.90)	0.78 (0.73, 0.84)	1.27 (1.20, 1.35)	0.68 (0.64, 0.73)	1.25 (1.18, 1.32)
Wealth index quintile	***	***	***	***	***
Poorest	1.20 (1.13, 1.27)	1.84 (1.69, 2.00)	0.41 (0.39, 0.44)	2.01 (1.86, 2.18)	0.44 (0.41, 0.47)
Poorer	1.15 (1.09, 1.21)	1.57 (1.45, 1.69)	0.54 (0.51, 0.57)	1.66 (1.54, 1.79)	0.58 (0.54, 0.61)
Middle	1.15 (1.10, 1.21)	1.37 (1.28, 1.48)	0.69 (0.65, 0.72)	1.43 (1.33, 1.53)	0.72 (0.68, 0.76)
Richer	1.09 (1.04, 1.14)	1.21 (1.13, 1.30)	0.81 (0.77, 0.85)	1.23 (1.15, 1.31)	0.85 (0.81, 0.89)
Richest	1.00 (ref)	1.00 (ref)	1.00 (ref)	1.00 (ref)	1.00 (ref)
Social group	***		***	***	***
Scheduled caste	1.09 (1.04, 1.14)	1.07 (1.01, 1.14)	0.89 (0.85, 0.93)	1.15 (1.08, 1.21)	0.97 (0.92, 1.02)
Scheduled tribe	1.34 (1.27, 1.42)	0.99 (0.92, 1.07)	0.81 (0.75, 0.86)	1.42 (1.32, 1.52)	0.98 (0.92, 1.05)
OBC	0.99 (0.95, 1.02)	1.08 (1.02, 1.13)	0.92 (0.88, 0.95)	1.07 (1.02, 1.13)	0.87 (0.83, 0.91)
Other caste, no caste or unsure	1.00 (ref)	1.00 (ref)	1.00 (ref)	1.00 (ref)	1.00 (ref)
Number of household members		***	***	***	
3 or fewer	1.00 (ref)	1.00 (ref)	1.00 (ref)	1.00 (ref)	1.00 (ref)
4 or 5	1.04 (1.00, 1.08)	1.10 (1.04, 1.16)	0.97 (0.93, 1.01)	1.14 (1.08, 1.20)	0.96 (0.92, 1.00)
6 or more	1.04 (1.00, 1.08)	1.16 (1.09, 1.23)	0.93 (0.89, 0.97)	1.19 (1.13, 1.25)	0.93 (0.89, 0.98)
Household owns agricultural land	***		***		
No	1.00 (ref)	1.00 (ref)	1.00 (ref)	1.00 (ref)	1.00 (ref)
Yes	1.04 (1.02, 1.07)	1.05 (1.01, 1.09)	0.93 (0.90, 0.96)	1.04 (1.01, 1.08)	0.96 (0.93, 0.99)
Marital status	***	***	***		***
Never in union	0.91 (0.87, 0.95)	1.28 (1.20, 1.36)	0.64 (0.60, 0.68)	1.03 (0.97, 1.09)	0.58 (0.55, 0.62)
Married	1.00 (ref)	1.00 (ref)	1.00 (ref)	1.00 (ref)	1.00 (ref)
Divorced/separated/widowed	1.09 (1.02, 1.15)	1.01 (0.91, 1.12)	0.85 (0.80, 0.91)	1.10 (1.01, 1.19)	0.92 (0.86, 0.99)
Region	***	***	***	***	***
Northern	1.00 (ref)	1.00 (ref)	1.00 (ref)	1.00 (ref)	1.00 (ref)
North Eastern	1.03 (0.96, 1.10)	0.68 (0.62, 0.75)	0.90 (0.84, 0.98)	0.82 (0.75, 0.91)	0.98 (0.90, 1.07)
Central	0.78 (0.75, 0.82)	0.96 (0.90, 1.02)	1.02 (0.97, 1.07)	0.77 (0.72, 0.82)	0.83 (0.78, 0.88)
Eastern	1.36 (1.28, 1.45)	1.07 (0.99, 1.16)	1.01 (0.94, 1.08)	1.40 (1.30, 1.51)	1.53 (1.42, 1.65)
Western	1.07 (1.00, 1.15)	1.70 (1.56, 1.85)	0.87 (0.81, 0.94)	1.82 (1.66, 2.00)	1.01 (0.93, 1.10)
Southern	0.86 (0.81, 0.92)	1.29 (1.18, 1.41)	1.42 (1.32, 1.52)	1.17 (1.07, 1.27)	1.38 (1.27, 1.49)
Religion		***	***	***	***
Hindu	1.00 (ref)	1.00 (ref)	1.00 (ref)	1.00 (ref)	1.00 (ref)
Muslim	0.98 (0.93, 1.03)	0.93 (0.87, 0.99)	1.17 (1.11, 1.24)	0.85 (0.79, 0.91)	1.09 (1.03, 1.16)
Other	0.88 (0.83, 0.95)	0.82 (0.75, 0.90)	1.23 (1.14, 1.33)	0.84 (0.77, 0.92)	1.13 (1.04, 1.23)
Adolescent birth (<20 years)			***	***	***
No	1.00 (ref)	1.00 (ref)	1.00 (ref)	1.00 (ref)	1.00 (ref)
Yes	1.00 (0.97, 1.03)	0.95 (0.91, 1.00)	1.07 (1.03, 1.10)	0.93 (0.90, 0.97)	1.09 (1.05, 1.13)
Short birth interval (<24 months)			***		
No	1.00 (ref)	1.00 (ref)	1.00 (ref)	1.00 (ref)	1.00 (ref)
Yes	0.96 (0.93, 0.99)	1.02 (0.97, 1.07)	1.06 (1.03, 1.10)	0.99 (0.94, 1.03)	1.04 (1.00, 1.07)
Cluster-level factors					
Change in annual number of hot days	***		***		***
Little change (<5 days more than average)	1.00 (ref)	1.00 (ref)	1.00 (ref)	1.00 (ref)	1.00 (ref)
More hot days (5 to 10 days more than average)	0.88 (0.85, 0.91)	1.07 (1.02, 1.12)	0.90 (0.86, 0.94)	0.99 (0.94, 1.04)	0.82 (0.78, 0.86)
Many more hot days (>10 days more than average)	0.90 (0.87, 0.94)	0.99 (0.94, 1.05)	0.80 (0.76, 0.83)	0.95 (0.89, 1.00)	0.74 (0.70, 0.78)
Change in annual precipitation intensity					
Much less precipitation (< -200mm less than average)	1.00 (0.89, 1.12)	0.99 (0.84, 1.16)	1.19 (1.04, 1.37)	0.93 (0.78, 1.09)	1.19 (1.00, 1.41)
Less precipitation (-100mm to -200mm less than average)	1.05 (1.00, 1.11)	1.01 (0.94, 1.09)	0.99 (0.93, 1.05)	1.03 (0.96, 1.11)	1.11 (1.04, 1.18)
Little change (-100mm to +100mm change)	1.00 (ref)	1.00 (ref)	1.00 (ref)	1.00 (ref)	1.00 (ref)
More precipitation (> 100mm more than average)	1.08 (1.03, 1.14)	0.95 (0.89, 1.02)	1.04 (0.98, 1.10)	1.08 (1.01, 1.16)	1.05 (0.98, 1.12)
SPEI (drought) index	***		***	***	***
First tertile (little change)	1.00 (ref)	1.00 (ref)	1.00 (ref)	1.00 (ref)	1.00 (ref)
Second tertile (drier than average)	1.02 (0.98, 1.06)	1.02 (0.97, 1.07)	1.00 (0.96, 1.05)	1.06 (1.01, 1.11)	1.00 (0.95, 1.05)
Third tertile (drier than average)	1.13 (1.07, 1.18)	1.03 (0.97, 1.10)	1.12 (1.06, 1.18)	1.17 (1.10, 1.25)	1.18 (1.11, 1.26)
Malaria incidence	***			***	
Low transmission	1.00 (ref)	1.00 (ref)	1.00 (ref)	1.00 (ref)	1.00 (ref)
Moderate transmission	1.04 (0.98, 1.09)	1.03 (0.97, 1.10)	0.94 (0.88, 1.00)	1.08 (1.01, 1.16)	0.96 (0.89, 1.04)
High transmission	1.10 (1.05, 1.16)	1.11 (1.04, 1.19)	0.93 (0.88, 0.99)	1.26 (1.18, 1.34)	1.00 (0.93, 1.07)

*** indicates significant joint Wald test at the Bonferroni-corrected threshold (p <0.00179).

This table presents odds ratios and 95% CIs from a multilevel multinomial logistic regression model. The baseline is normal weight without anaemia (no burden of malnutrition) in all outcomes (N = 120,285). Pregnant women and those up to 3 months post-partum were excluded from analysis. Counts presented are unweighted. Anaemia: haemoglobin concentration <12.0g/dL. Underweight: BMI <18.5kg/m2. Overweight/obesity: BMI >23kg/m2.

BMI, body mass index; BPL, below poverty line; NFHS, National Family and Health Survey; SPEI, Standardised Precipitation Evapotranspiration Index.

Proximal factors largely did not show strong associations with the double burdens, with the exception of tobacco consumption which was associated with 63% higher odds of underweight-anaemia (adjusted OR (aOR)=1.63 (95% CI 1.51 to 1.75)). A unit increase in the iron-rich food consumption index was significantly associated with 2% lower odds of underweight-anaemia (aOR=0.98 (0.97–0.99)). A vegetarian diet was associated with 8% higher odds of overweight/obesity-anaemia (aOR=1.08 (1.04–1.13)) and 7% higher odds of underweight-anaemia (aOR=1.07 (1.03–1.11)). Alcohol and daily unhealthy food consumption were not significantly associated with either double burden.

There was some evidence that the intermediate healthcare, WASH and social protection service access variables were associated with the double burdens, although effect sizes were not large. Lack of healthcare providers or drugs present at health facilities being ‘a big concern’ (aOR=1.06 (1.03–1.10)) was associated with higher odds of underweight-anaemia, while modern contraceptive use was associated with lower odds (aOR=0.87 (0.83–0.90)). WASH and health access variables were found to be significantly associated with overweight/obesity-anaemia, with health insurance coverage (aOR=0.92 (0.89–0.96)), unimproved toilet facility (aOR=0.90 (0.86–0.95)) and BPL card possession (aOR=0.93 (0.90–0.97)) associated with lower odds of overweight/obesity-anaemia. Associations with intermediate risk factors (healthcare, WASH and social protection service access variables) observed in univariable analyses mostly attenuated or disappeared after adjustment for distal factors.

Distal factors, particularly sociodemographic variables, were strongly associated with both double burden outcomes, often in different directions. Variables such as lower education and wealth quintile, younger age, higher number of household members, belonging to a disadvantaged social group and Hindu religion were associated with higher odds of having underweight-anaemia (aOR between 1.07 and 2.01; see table 3) and lower odds of overweight/obesity-anaemia (aOR between 0.98 and 0.44; see table 3). Having had an adolescent birth was associated with modestly but significantly lower odds of underweight-anaemia (aOR=0.93 (0.90–0.97)) and higher odds of overweight/obesity-anaemia (aOR=1.09 (1.05–1.13)), while short birth intervals were not significantly associated with either double burden. There was high variability in associations seen for different regions of India, with the Western region associated with the highest odds of underweight-anaemia (aOR=1.82 (1.66–12.00); reference: Northern region) and the Eastern region was associated with the highest odds of overweight/obesity-anaemia (aOR=1.53 (1.42–1.64); reference: Northern region); see table 3).

Where associations with environmental variables were present, effect sizes tended to be moderate. Women living in areas which had experienced more hot days in the last 10 years than the 1950–2000 average had significantly lower odds of overweight/obesity-anaemia (aOR=0.74 (0.70–0.78)), but no significant association was observed with underweight-anaemia. No significant association was observed for either double burden with living in an area experiencing change in the intensity of annual precipitation than the 1950–2000 average. Living in an area experiencing the highest tertile of SPEI index (areas drier than the long-term average) was associated with higher odds of both underweight-anaemia (aOR=1.17 (1.10–1.25)) and overweight-anaemia (aOR=1.18 (1.11, 1.26)). Living in an area with higher malaria incidence was significantly associated with higher odds of underweight-anaemia (high transmission aOR=1.26 (1.18–1.34)) but was not associated with overweight/obesity-anaemia.

### Risk factors for the single burdens in 2019–2021

Similarly to the double burdens, for the single burdens the larger effect sizes were found among the distal sociodemographic variables. Directions of associations of these variables commonly diverged for underweight only and overweight/obesity only outcomes. The associations of distal variables with anaemia only were usually consistent in direction with underweight only. Scheduled tribe background and region were particularly strongly associated with anaemia only.

Other than tobacco use, no proximal or intermediate factors were associated with anaemia only. The direction of association of proximal and intermediate variables for underweight only or overweight/obesity only was largely consistent with the associations described for the double burdens, but attainment of statistical significance varied (see table 3).

Living in an area experiencing a higher number of hot days compared with the 1950–2000 average was associated with lower odds of the anaemia only and overweight/obesity only outcomes. Living in an area experiencing the highest tertile of SPEI index (areas drier than the long-term average) was associated with higher odds of the anaemia only and overweight/obesity only outcomes. Living in an area with high transmission of malaria was associated with higher odds of anaemia only but not other outcomes.

### Receiver operating characteristic curves

**Figure 5:**
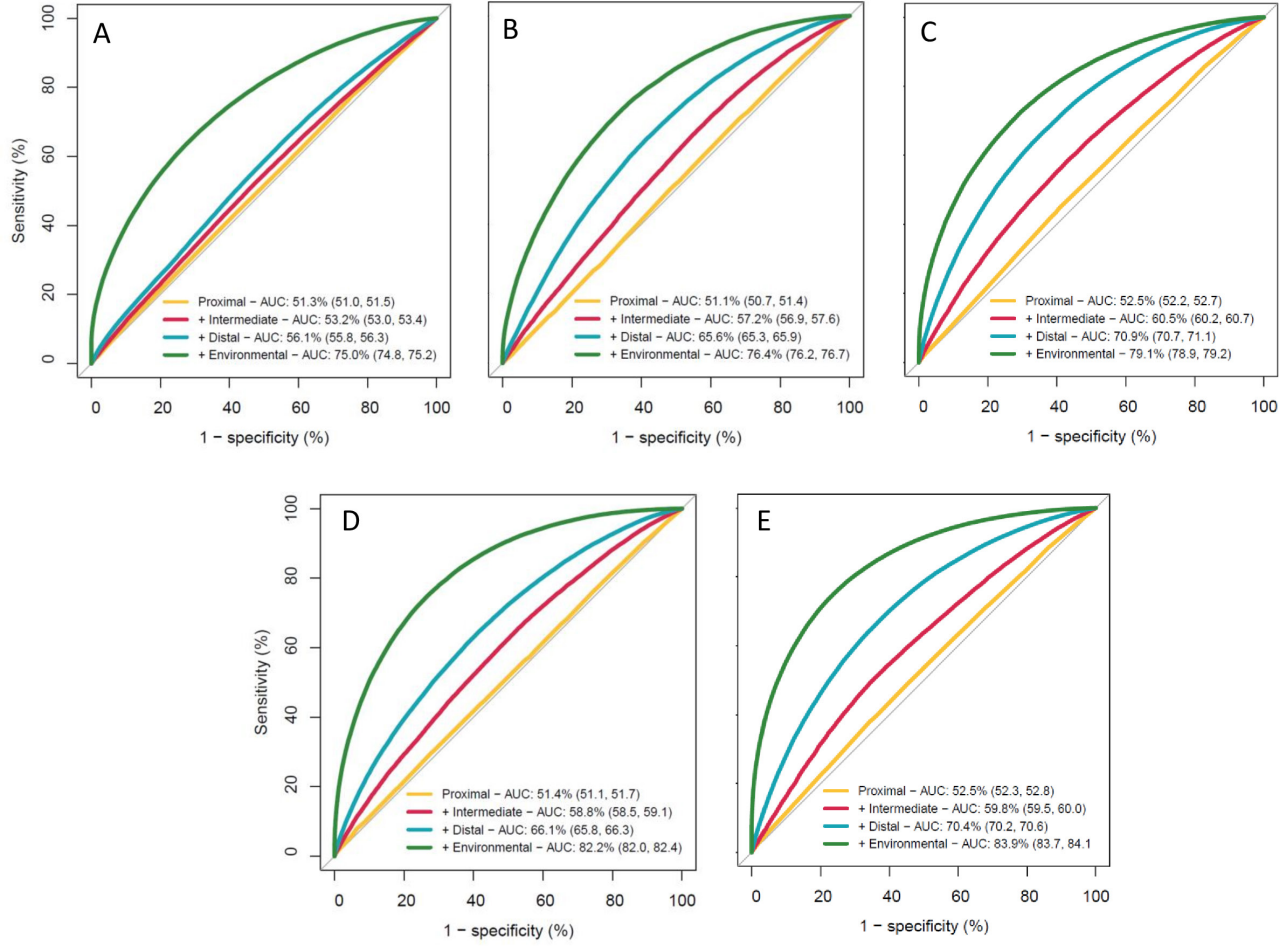
Receiver operating curve and AUC statistics for multivariable logistic regression model with five binary outcomes after progressive adjustment for groups of potential risk factors**. A**) Anaemia only. (**B**) Underweight only. (**C**) Overweight/obesity only. (**D**) Underweight-anaemia double burden. (**E**) Overweight/obesity-anaemia double burden. Normal weight without anaemia is the common baseline outcome in all binary outcomes. Notes: ‘Proximal’ includes dietary and lifestyle variables; ‘Intermediate’ includes health, water, sanitation and hygiene, and social protection service access variables; ‘Distal’ includes sociodemographic and reproductive variables; and ‘Environmental’ includes climatic and environmental variables. Refer to figure 2 for a full list of variables and groupings. AUC, area under the curve.

Figure 5 shows that across all outcomes, the predictive performance of the model when adjusting for only individual-level factors was low, but additionally adjusting for environmental factors substantially increased the AUC statistic to above the conventional threshold for clinical utility of a predictive model (AUC ≥75%). This was particularly true for the anaemia only outcome (figure 5a). The model performed best for double burden outcomes, with AUC statistics exceeding 80%.

### Sensitivity analysis: moderate/severe anaemia

Re-running the primary analysis with a lower threshold for defining anaemia (corresponding to classification with moderate/severe anaemia) did not differ substantively from results observed using the conventional higher threshold. Results for the sensitivity analysis are presented in the supplementary materials (online supplemental Table 5).

### Secondary analysis: risk factors for the double burdens over time

Significant differences were found between the strength of associations of determinants identified for the 2019–2021 data and those for previous survey rounds (2005–2006 and 2015–2016) for the multiple group analysis conducted using variables available in all survey rounds. Among the most prominent were differences in strength of association for older age, urban residence, higher wealth quintiles and region (see table 4table 4).

**Table 4:** Associations in multiple survey rounds of potential risk factors with the single and double burdens of malnutrition compared to no malnutrition burden: NFHS-4 (2015–2016)

NFHS-4, 2015–2016	Single burdens			Double burdens	
	OR (95% CI)			OR (95% CI)	
	Anaemia only	Underweight only	Overweight/obesity only	Underweight-anaemia	Overweight/obesity-anaemia
N =620 029	161 104	52 367	102 463	78 725	94 346
Proximal factors					
Iron-rich food consumption index	***	***	***	***	
	0.99 (0.98, 0.99)	0.97 (0.96, 0.98)	1.02 (1.01, 1.03)	0.97 (0.96, 0.98)	1.01 (1.00, 1.02)
Vegetarian					
No	1.00 (ref)	1.00 (ref)	1.00 (ref)	1.00 (ref)	1.00 (ref)
Yes	0.98 (0.95, 0.98)	1.05 (1.01, 1.09)	1.01 (0.97, 1.05)	1.01 (0.97, 1.05)	0.98 (0.95, 1.02)
Drinks alcohol					
No	1.00 (ref)	1.00 (ref)	1.00 (ref)	1.00 (ref)	1.00 (ref)
Yes	0.89 (0.82, 0.97)	0.94 (0.83, 1.06)	0.96 (0.85, 1.08)	0.91 (0.82, 1.01)	0.89 (0.77, 1.02)
Uses tobacco	***	***	***	***	***
No	1.00 (ref)	1.00 (ref)	1.00 (ref)	1.00 (ref)	1.00 (ref)
Yes	1.07 (1.03, 1.12)	1.43 (1.35, 1.51)	0.84 (0.80, 0.89)	1.51 (1.44, 1.58)	0.86 (0.82, 0.91)
Intermediate factors					
Has health insurance					
No	1.00 (ref)	1.00 (ref)	1.00 (ref)	1.00 (ref)	1.00 (ref)
Yes	1.01 (0.97, 1.04)	1.00 (0.96, 1.04)	1.05 (1.01, 1.09)	0.99 (0.95, 1.03)	1.02 (0.98, 1.06)
Difficulty with distance to health facility				***	
Not a big concern	1.00 (ref)	1.00 (ref)	1.00 (ref)	1.00 (ref)	1.00 (ref)
Big concern	1.04 (1.01, 1.06)	1.01 (0.98, 1.04)	1.00 (0.97, 1.04)	1.06 (1.03, 1.10)	1.05 (1.01, 1.08)
Concern no providers or drugs at health facility					***
Not a big concern	1.00 (ref)	1.00 (ref)	1.00 (ref)	1.00 (ref)	1.00 (ref)
Big concern	0.97 (0.95, 1.00)	1.01 (0.98, 1.05)	0.96 (0.94, 0.99)	0.98 (0.95, 1.01)	0.93 (0.90, 0.96)
Modern contraceptive use		***	***	***	
No	1.00 (ref)	1.00 (ref)	1.00 (ref)	1.00 (ref)	1.00 (ref)
Yes	1.00 (0.97, 1.02)	0.90 (0.86, 0.93)	0.94 (0.91, 0.97)	0.89 (0.86, 0.92)	1.02 (0.99, 1.06)
Unimproved drinking water					***
No	1.00 (ref)	1.00 (ref)	1.00 (ref)	1.00 (ref)	1.00 (ref)
Yes (unimproved)	1.03 (0.99, 1.08)	0.99 (0.94, 1.04)	0.95 (0.90, 1.00)	1.04 (0.99, 1.10)	0.85 (0.80, 0.90)
Unimproved toilet facility			***	***	***
No	1.00 (ref)	1.00 (ref)	1.00 (ref)	1.00 (ref)	1.00 (ref)
Yes (unimproved)	0.99 (0.96, 1.02)	1.04 (1.00, 1.09)	0.89 (0.86, 0.93)	1.06 (1.02, 1.10)	0.86 (0.82, 0.90)
Has BPL card		***	***	***	***
No	1.00 (ref)	1.00 (ref)	1.00 (ref)	1.00 (ref)	1.00 (ref)
Yes	1.01 (0.98, 1.03)	1.09 (1.06, 1.12)	0.89 (0.87, 0.92)	1.10 (1.06, 1.14)	0.93 (0.90, 0.96)
Distal factors					
Age	***	***	***	***	***
15–19	1.07 (1.02, 1.13)	1.31 (1.23, 1.39)	0.42 (0.39, 0.45)	1.33 (1.25, 1.41)	0.46 (0.43, 0.50)
20–24	1.04 (1.00, 1.09)	1.29 (1.22, 1.36)	0.61 (0.58, 0.64)	1.32 (1.26, 1.39)	0.64 (0.60, 0.67)
25–29	1.00 (ref)	1.00 (ref)	1.00 (ref)	1.00 (ref)	1.00 (ref)
30–35	0.96 (0.92, 1.00)	0.80 (0.76, 0.85)	1.48 (1.40, 1.55)	0.82 (0.78, 0.86)	1.42 (1.35, 1.49)
35–39	1.02 (0.98, 1.07)	0.71 (0.67, 0.75)	1.80 (1.72, 1.89)	0.74 (0.70, 0.78)	1.84 (1.75, 1.93)
40–45	0.99 (0.94, 1.04)	0.67 (0.63, 0.72)	2.10 (1.99, 2.21)	0.77 (0.72, 0.81)	2.25 (2.13, 2.37)
45–49	0.97 (0.92, 1.01)	0.63 (0.59, 0.67)	2.31 (2.20, 2.43)	0.71 (0.67, 0.76)	2.29 (2.18, 2.42)
Residence	***	***	***		***
Urban residence	1.00 (ref)	1.00 (ref)	1.00 (ref)	1.00 (ref)	1.00 (ref)
Rural residence	0.94 (0.90, 0.97)	1.10 (1.05, 1.15)	0.83 (0.80, 0.86)	1.03 (0.98, 1.08)	0.79 (0.76, 0.83)
Education	***	***	***	***	***
No education	1.00 (ref)	1.00 (ref)	1.00 (ref)	1.00 (ref)	1.00 (ref)
Incomplete primary	1.00 (0.95, 1.05)	0.91 (0.85, 0.97)	1.14 (1.07, 1.21)	0.94 (0.89, 1.00)	1.20 (1.12, 1.27)
Incomplete secondary	0.94 (0.92, 0.97)	0.82 (0.79, 0.85)	1.29 (1.24, 1.33)	0.79 (0.76, 0.82)	1.30 (1.25, 1.35)
Complete secondary and higher	0.84 (0.80, 0.88)	0.75 (0.71, 0.79)	1.14 (1.09, 1.21)	0.67 (0.64, 0.71)	1.14 (1.08, 1.20)
Wealth index quintile		***	***	***	***
Poorest	1.10 (1.04, 1.17)	1.75 (1.62, 1.90)	0.30 (0.28, 0.32)	1.86 (1.70, 2.04)	0.29 (0.27, 0.31)
Poorer	1.08 (1.02, 1.14)	1.58 (1.47, 1.69)	0.44 (0.42, 0.47)	1.68 (1.54, 1.84)	0.44 (0.41, 0.47)
Middle	1.09 (1.04, 1.15)	1.39 (1.30, 1.49)	0.61 (0.57, 0.64)	1.50 (1.38, 1.64)	0.61 (0.58, 0.65)
Richer	1.06 (1.02, 1.11)	1.21 (1.13, 1.29)	0.80 (0.76, 0.84)	1.32 (1.22, 1.42)	0.81 (0.77, 0.85)
Richest	1.00 (ref)	1.00 (ref)	1.00 (ref)	1.00 (ref)	1.00 (ref)
Social group	***		***	***	***
Schedule caste	1.09 (1.04, 1.13)	1.08 (1.02, 1.14)	0.88 (0.84, 0.92)	1.19 (1.13, 1.26)	1.03 (0.98, 1.08)
Schedule tribe	1.33 (1.27, 1.40)	0.99 (0.93, 1.06)	0.80 (0.75, 0.85)	1.58 (1.49, 1.67)	0.99 (0.93, 1.06)
OBC	0.97 (0.94, 1.00)	1.05 (1.01, 1.10)	0.87 (0.84, 0.90)	1.07 (1.03, 1.11)	0.88 (0.85, 0.92)
Other caste, no caste or unsure	1.00 (ref)	1.00 (ref)	1.00 (ref)	1.00 (ref)	1.00 (ref)
Number of household members	***	***	***	***	
3 or fewer	1.00 (ref)	1.00 (ref)	1.00 (ref)	1.00 (ref)	1.00 (ref)
4 or 5	1.01 (0.97, 1.05)	1.12 (1.06, 1.18)	0.92 (0.89, 0.96)	1.13 (1.08, 1.18)	0.95 (0.91, 0.99)
6 or more	1.06 (1.02, 1.10)	1.16 (1.10, 1.22)	0.88 (0.85, 0.92)	1.19 (1.14, 1.24)	0.93 (0.89, 0.97)
Household ownership of land			***		***
No	1.00 (ref)	1.00 (ref)	1.00 (ref)	1.00 (ref)	1.00 (ref)
Yes	1.01 (0.99, 1.04)	1.03 (1.00, 1.06)	0.87 (0.84, 0.89)	1.01 (0.98, 1.04)	0.90 (0.88, 0.93)
Marital status	***	***	***	***	***
Never in union	0.93 (0.89, 0.97)	1.23 (1.17, 1.30)	0.66 (0.62, 0.69)	1.07 (1.01, 1.12)	0.64 (0.60, 0.68)
Married	1.00 (ref)	1.00 (ref)	1.00 (ref)	1.00 (ref)	1.00 (ref)
Divorced/separated/widowed	1.10 (1.03, 1.17)	1.02 (0.94, 1.11)	0.84 (0.79, 0.90)	1.19 (1.11, 1.27)	0.96 (0.90, 1.03)
Region	***	***	***	***	***
Northern	1.00 (ref)	1.00 (ref)	1.00 (ref)	1.00 (ref)	1.00 (ref)
North Eastern	0.72 (0.68, 0.76)	0.96 (0.90, 1.03)	0.90 (0.85, 0.96)	0.61 (0.56, 0.67)	0.66 (0.62, 0.71)
Central	0.93 (0.90, 0.96)	1.02 (0.97, 1.07)	1.02 (0.98, 1.06)	0.89 (0.83, 0.96)	0.88 (0.84, 0.92)
Eastern	1.27 (1.22, 1.33)	1.09 (1.03, 1.16)	1.09 (1.03, 1.15)	1.32 (1.22, 1.43)	1.34 (1.26, 1.42)
Western	0.98 (0.92, 1.03)	1.56 (1.46, 1.66)	1.10 (1.04, 1.17)	1.45 (1.32, 1.59)	0.97 (0.91, 1.04)
Southern	1.11 (1.05, 1.17)	1.13 (1.06, 1.21)	1.39 (1.31, 1.47)	1.15 (1.05, 1.26)	1.41 (1.33, 1.50)
Religion	***	***	***	***	***
Hindu	1.00 (ref)	1.00 (ref)	1.00 (ref)	1.00 (ref)	1.00 (ref)
Muslim	0.91 (0.87, 0.95)	0.98 (0.93, 1.03)	1.20 (1.14, 1.25)	0.89 (0.84, 0.93)	1.14 (1.09, 1.20)
Other	1.00 (0.94, 1.06)	0.82 (0.76, 0.89)	1.16 (1.08, 1.23)	0.79 (0.72, 0.86)	1.05 (0.99, 1.13)
Adolescent birth (<20 years)			***		***
No	1.00 (ref)	1.00 (ref)	1.00 (ref)	1.00 (ref)	1.00 (ref)
Yes	1.00 (0.97, 1.02)	0.96 (0.92, 1.00)	1.10 (1.06, 1.13)	0.98 (0.95, 1.02)	1.13 (1.09, 1.17)
Short birth interval (<24 months)			***		***
No	1.00 (ref)	1.00 (ref)	1.00 (ref)	1.00 (ref)	1.00 (ref)
Yes	1.00 (0.97, 1.03)	1.05 (1.01, 1.10)	1.07 (1.03, 1.10)	1.02 (0.98, 1.05)	1.09 (1.06, 1.13)

*** indicates significant joint Wald test at the Bonferroni-corrected threshold (p <0.00217).

This table presents odds ratios and 95% CIs from a multilevel multinomial logistic regression model. The baseline is normal weight without anaemia (no burden of malnutrition) in all outcomes (n=131 024). Pregnant women and those up to 3 months post-partum were excluded from analysis. Counts presented are unweighted. Anaemia: haemoglobin concentration <12.0 g/dL. Underweight: BMI <18.5 kg/m2. Overweight/obesity: BMI >23 kg/m2.

BMI, body mass index; BPL, below poverty line; NFHS, National Family and Health Survey.

The effect size of the association was observed in 2005–2006 for overweight/obesity and its double burden, whereby increasing age was associated with higher odds of the outcomes, marginally reduced over time (overweight/obesity only: 15–19 years (2005–2006, aOR=0.37 (0.31–0.43); 2015–2016, aOR=0.42 (0.39–0.45); 2019–2021, aOR=0.44 (0.41–0.47)) and 45–49 years (2005–2006, aOR=2.58 (2.27–2.93); 2015–2016, aOR=2.31 (2.20–2.43); 2019–2021, 2.14 (2.03–2.25); ref: 25–29 years). In contrast, the effect size for the positive association of younger age with underweight became significant and increased over time (underweight only: 15–19 years (2005–2006 aOR=0.90 (0.79–1.02), 2015–2016 aOR=1.31 (1.23–1.39), 2019–2021 aOR=1.57 (1.46–1.67)); ref: 25–29 years).

The OR for rural compared with urban residence attenuated in more recent survey rounds for overweight/obesity-anaemia (2005–2006, aOR=0.71 (0.64–0.78); 2015–2016, aOR=0.79 (0.76–0.83); 2019–2021, aOR=0.92 (0.88–0.96)), but a commensurate change in the effect size of the association for underweight-anaemia was not seen.

The effect size of the association between lower wealth quintile and lower odds of overweight/obesity-anaemia attenuated in more recent survey rounds (lowest wealth quintile (ref highest): 2005–2006 a0R=0.27 (0.22–0.34); 2015–2016 aOR=0.29 (0.27–0.31), 2019–2021 aOR=0.43 (0.40–0.46)). However, this was not observed for underweight-anaemia (2005–2006 aOR=2.35 (2.00–2.74), 2015–2016 aOR=1.86 (1.70–2.04), 2019–2021 aOR=2.01 (1.86–2.16)).

OR values varied significantly across survey rounds for different regions for all outcomes, although direction of association was mostly maintained across surveys.

## Discussion

This study has shown that the prevalence of the double burdens was high among women of reproductive age in the study population, with approximately one in 10 having underweight-anaemia and one in five having overweight/obesity-anaemia in 2019–2021. The prevalence of underweight-anaemia had approximately halved between 2005–2006 and 2019–2021, while the prevalence of overweight/obesity-anaemia had more than doubled. These double burdens are distributed unevenly across India, such that some districts experience substantially higher prevalence. The strongest risk factors for both double burdens were sociodemographic, and, as expected, these variables commonly showed opposite directions of association with the two double burdens.

The prevalence of underweight-anaemia has rarely been reported in South Asia and was found in this study in India in 2019–2021 (11%) to be slightly lower than has been identified in Bangladesh (14%).^54^ The prevalence of overweight/obesity-anaemia in 2019–2021 (21%) was higher than has been found in recent DHS rounds in neighbouring Nepal (7%),^20^ Myanmar (10%)^20^ and Pakistan (15%)^29^ and similar to levels observed in Afghanistan (19%),^29^ although it should be noted that the present analysis used a lower BMI threshold for defining overweight/obesity. This analysis used Asian BMI cut-offs (BMI ≥23 kg/m^2^) and identified double the prevalence of overweight/obesity-anaemia in India in 2012–2021 compared with previous studies using the higher WHO cut-offs (BMI ≥25 kg/m^2^) in the same NFHS-5 dataset.^18 20^ Justification for using Asian cut-offs in South Asian populations is compelling.^22^,^24^ Their use in this study helps reveal the full scale of the overweight/obesity-anaemia public health problem facing India.

Anaemia prevalence has remained stagnant in India since 2005–2006, affecting more than half of reproductive-age women in all survey rounds, despite the scale-up of national anaemia reduction initiatives. During the 15 years between 2005–2006 and 2019–2021 surveys, the prevalence of underweight declined by 50% while that of overweight/obesity increased by more than 75%. Changes in the prevalence of the double burdens are hence driven by these substantial changes in BMI among reproductive-age women. Anaemia, which has remained stagnant with a high population-level prevalence, is common in both under-nourished and over-nourished women, meaning that both inadequate and excessive energy balances are coupled with insufficient micronutrient intake in these populations. This has important implications for how services deliver dietary messages and supports the integration of messaging on healthy eating patterns that promote micronutrient sufficiency, going beyond a focus on body weight or caloric intake.

Mapping indicated that the underweight-anaemia and overweight/obesity-anaemia double burdens were prevalent, distributed similarly to BMI overall in India, and largely spatially distinct. Despite substantial changes in the prevalence of overall underweight and overweight/obesity between 2005–2006 and 2019–2021, the areas most affected by these burdens remained similar across survey rounds. The spatial distinctness of the two double burdens displayed in figure 3 reflects both the known spatial inequalities in wealth distribution in India and the strong socioeconomic risk factors for the double burdens identified in this study. The underweight-anaemia burden is concentrated in a band of districts across central and inland eastern India where poverty is also clustered,^55^ and districts in this band have the lowest prevalence of overweight/obesity-anaemia. National-level policy must address different double burden challenges in different areas, but current national universal delivery programmes are not designed to specifically respond to these spatial disparities.

This study was able to corroborate the strong associations of overweight/obesity-anaemia with sociodemographic variables identified in previous studies in South Asia, including for urban residence^20 21 28 29^ and increasing age,^18 20 21 25 29^ wealth^1820 21 25 27^,^29^ and education.^18 20 25 29^ This study confirms that associations with these factors, alongside other sociodemographic risk factors including social group, region and marital status, remain strong after adjustment for a broad range of service access, diet and lifestyle variables. Associations for overweight/obesity-anaemia were similar to those observed for overweight/obesity only. The strong concentration of overweight/obesity among certain sociodemographic groups has been found consistently in studies in India and variously attributed to poor opportunities for physical activity and a higher proportion of energy intake from fats among higher socioeconomic populations.^56 57^ These factors potentially highlight elements of nutritional intake and cultural norms that the other variables in our analysis were not able to capture.

Providing the first estimates of risk factors for underweight-anaemia in South Asia, the study finds that tobacco use, non-use of modern contraception, region, age under 25 (vs age 25–29), lower education or wealth, scheduled tribe or caste background, living in a household with more than three members, having Hindu faith (vs Muslim or other) and living in areas with increased precipitation intensity (>100 mm more in highest intensity periods) or high malaria transmission were all associated with 15% or higher odds of the burden, while most proximal and intermediate factors showed no or weak association after full adjustment. The associations of underweight-anaemia with lower socioeconomic status,^58 59^ contraceptive non-use,^58 59^ tobacco,^60^ region^61^ and young age^58 59 61^ are consistent with known risk factors for underweight in South Asia, and our results for the underweight only outcome. The association of underweight-anaemia with scheduled tribe background is likely in part driven by the high burden of sickle-cell anaemia in this population, reported as over 50% in certain groups.^62^ The lack of association with short birth interval is corroborated by Rahman *et al*’s findings in Bangladesh,^54^ but higher precision in this study’s estimates increases confidence in this finding. However, associations observed with environmental variables are novel and require careful interpretation.

The ROC curves demonstrated that environmental factors make a large difference in the predictive performance of the model, even after adjustment for 24 other individual-level factors. This analysis does not find evidence that the associations with malnutrition outcomes observed for environmental variables were explained through access to healthcare, WASH or social protection services, diet, lifestyle, and sociodemographic or reproductive history variables. Previous studies suggest that high temperatures, drought conditions and changing rainfall patterns can lead to lower crop yields, lower nutrient content of cereals, food insecurity and shifts towards consumption of packaged foods and less nutritious diets as a result of these changes.^47 63 64^ This analysis did not include these mediating food system variables and so cannot verify this pathway of effect, but further work investigating these hypotheses is merited. Whether or not the environmental associations observed are causal, the environmental variables describe areas of India with higher levels of the double burdens after controlling for individual-level factors, and hence can potentially inform spatial targets for programmes.

This study is the first exploratory analysis assessing the risk factors for the underweight-anaemia double burden in South Asia, to the best of our knowledge. It is also one of the few global studies which assesses risk factors for the double burdens beyond a select group of key sociodemographic variables, analysing all single and double burdens simultaneously with a cohesive baseline group, and assessing changes in risk factor effect sizes over time. However, this work has some limitations. First, one cannot exclude the presence of residual confounding for associations of environmental and sociodemographic factors as discussed above. Second, 4.9% of women interviewed refused or had invalid BMI or anaemia measurements (online supplemental Table 2), with this group significantly more likely to be urban, richer, better educated and from central or northern regions. This may introduce some selection bias, although not likely to have a significant impact on the findings due to the large sample size of the included population. Third, the large number of risk factors analysed is a strength of this study but presents risks associated with multiple testing and chance findings that remain possible despite the conservative Bonferroni correction criteria. Finally, the cluster-level survey weights used are approximations of the true sample weights. Different weighting approaches investigated show variation in OR estimates that are further from the null for environmental factors. Further discussion in the supplementary materials discusses why such variability is unlikely to change the conclusions presented here.

Despite rising awareness of these double burdens, programmes addressing malnutrition remain largely siloed in India. The government’s flagship Anaemia Mukt Bharat (Anaemia Free India) programme provides all reproductive-age women (15–49 years) with prophylactic Iron and Folic Acid supplementation. Interlinked strategies support screening and treatment of non-nutritional anaemias in endemic pockets.^62^ To combat underweight, various programmes under different ministries provide nutrition supplementation to pregnant and lactating mothers.^65^ Overweight/obesity prevention strategies are similarly distributed across government schemes, which include food advertising and labelling regulation, healthy lifestyle promotion and screening of chronic diseases associated with overweight. ^66^Common to these strategies is an emphasis on universal programming in which services are delivered to broadly specified demographic groups.

The effects of programmes that address only one part of the double burden (underweight, overweight/obesity, anaemia) on other parts of the individual’s nutritional status are not well understood. Calls for designing ‘double-duty’ programmes which address two or more forms of malnutrition concurrently have been growing.^1^ The high levels of concurrent anaemia with underweight and overweight/obesity warrant that programmes consider holistic service delivery that, for example, acknowledges that women in receipt of services to reduce overweight and obesity may not only need support to decrease overall calorie intake but also to increase nutrient-dense food intake. Risk factor associations for the double burdens were largely consistent with those for BMI only, suggesting that programmes seeking to particularly target the double burdens could be patterned after or integrated with underweight and overweight/obesity targeting programmes, with anaemia reduction as a concurrent aim across all services.

The double burdens are prevalent in India and show distinct spatial and sociodemographic patterning, as is consistent with the distributions of BMI in India. While previous studies had only investigated sociodemographic risk factors for the double burdens in South Asia, this study confirms that after adjustment for 28 environmental, sociodemographic, service access and diet and lifestyle variables available in the DHS, sociodemographic factors do remain the strongest risk factors for both double burdens, with associations with proximal diet/lifestyle and intermediate service access variables attenuating or disappearing after adjustment for sociodemographic characteristics. Over the 2005–2006 to 2019–2021 period, younger age has become increasingly strongly associated with underweight-anaemia, suggesting an increasing opportunity to target services among this high-risk group. Concurrent to this trend, while associations of overweight/obesity-anaemia with higher wealth quintile and older age remain among the strongest between 2005–2006 and 2019–2021, these associations have weakened as the characteristics of individuals with overweight/obesity have diversified. Policy must keep abreast of these evolving obesogenic landscapes and increasingly target broader swathes of the population.

While malnutrition programmes in India currently provide non-targeted interventions across most women of reproductive age, this study suggests that the distinct spatial characteristics and strong sociodemographic risk factors for the two double burdens warrant further investigation of the merits of including spatial or high-risk individual targeting of existing malnutrition programmes to improve efficiency and address the distinct sources of malnutrition in different populations.

## Supplementary material

10.1136/bmjph-2025-002730 online supplemental file 1

## Data Availability

This study used data from the Indian National Family Health Survey round 3 (2005-06), round 4 (2015-16) and round 5 (2019-21). These datasets are made publicly available for researchers from the Demographic and Health Surveys (DHS) program at https://dhsprogram.com/. Environmental variables were derived from data that is publicly available in the Data Catalog of the World Bank Climate Change Knowledge Portal at https://climateknowledgeportal.worldbank.org/download-data.
