# Supplementary material for: Risk factors for the intra-individual double burdens of malnutrition among reproductive-age women in India: a secondary analysis of three rounds of the National Family and Health Survey
Source: BMJ Public Health. 2025 Oct 23;3(2):e002730. doi: 10.1136/bmjph-2025-002730 (PMC12557752; doi:10.1136/bmjph-2025-002730)
Supplement: online supplemental file 1 [file bmjph-3-2-s001.docx]

**Risk factors for the intra-individual double burdens of malnutrition among reproductive-age women in India: a secondary analysis of three rounds of the NFHS**

**Supplementary materials**

**Further methods**

Search strategy for scoping review to inform variable selection

The aim of the scoping review was to appraise the current evidence of risk factors for the intra-individual double burdens of co-occurring anaemia with underweight or overweight/obesity in non-pregnant women of reproductive age in South Asia. MEDLINE and Embase databases were searched, and their results were supplemented with the first 100 entries obtained from a web search using Google Scholar to identify reports published in non-medical journals, and a further manual search using Google Scholar and screening of reference lists.

Reports were eligible for review if they analysed either intra-individual double burden (using any measure of adiposity status and definition for underweight or overweight) among women between ages 15-49 in any of the eight South Asian countries as defined by the World Bank.^1^ Reports were excluded if the main focus of the study was pregnant women or maternal outcomes or if the report did not present quantitative outcomes or estimated only prevalences of the double burdens.

The systematic search was conducted on the 17th of May 2024. MEDLINE, Embase and Google Scholar were searched for terms associated with adiposity and its measures, anaemia, female, risk factors, co-occurrence or double burden, and South Asia and its constituent countries. Full search strategies are presented in Supplementary Table 9. The search was limited to reports published in the last 10 years (2014 onwards) because the prevalences and characteristics of underweight and overweight/obesity in the region have seen large shifts in the last decade alone.^2^ There were no language restrictions.

Framework for variable identification and selection

The intra-individual double burden has no well-established conceptual framework that maps the causal or associational pathways between risk factors and intra-individual double burden of malnutrition outcome. However, risk factors for the single burdens of underweight, overweight/obesity and anaemia are well established and existing conceptual frameworks based on extensive literature review have been developed and used widely in practice. In order to develop a conceptual framework for the double burdens, this study hence draws together six frameworks for the individual single burdens and supplements them with findings from the scoping review of risk factors for the double burden of malnutrition.

For underweight, the UNICEF 2020 framework for Maternal and Child Nutrition^1^ and the UNICEF 2013 framework for Maternal and Child Undernutrition^2^ were used. For overweight/obesity, the Foresight 2007 Obesity System Map,^3^ the ROOTS obesity framework,^4^ and the UNICEF 2020 framework for Maternal and Child Nutrition^1^ were used. For anaemia, the Balarajan et al., 2011^5^ and WHO accelerating anaemia reduction^6^ frameworks were used. These frameworks are combined in Supplementary Table 2, with determinants across all frameworks synthesised under common labels, and available DHS variables then mapped to these synthesised labels. Some variables are available in the NFHS-5 and other DHS surveys, but are not available for the full sample of reproductive-age women in the NFHS-5. They are included in Supplementary Table 2 and are underlined, but are not carried forward to the conceptual framework presented in Figure 1.

The UNICEF 2020 framework was used as a guide to structuring of Figure 1 into environmental, distal, intermediate and proximal categories.

Sampling design and calculation of multilevel weightings

Environmental variables were constant within clusters, which correspond to a village in rural areas or census enumeration block in urban areas. The multivariable analysis performed hence estimated the effects of both cluster-level and individual-level factors on individual outcomes. To accurately do so using complex survey data, a multilevel model must be specified for which both individual-level and cluster-level survey weights are necessary.

The DHS provides household and woman-level survey weights which adjust each observation for their likelihood of being sampled. A standard process for calculating the corresponding cluster-level weights (the inverse probability of each cluster being selected for sampling) from the provided women’s and household weights is provided by the DHS and uses some additional data about the sampling units which is usually provided by the DHS or otherwise publicly available. DHS statisticians have confirmed that the data necessary for following the standard method for the Indian DHS 2019-21 (NFHS-5) are not available and may have been lost.^7^

In order to still specify a multilevel model with adequate adjustment for differences in probability of sampling at the cluster level, this study estimates the cluster-level weight by using the household-level weight with reverted adjustment for household non-response at the state level.

While this method does not revert adjustment for different probability of household selection within a cluster based on cluster size (varying between 40 and 300 households), assessment of publicly available data from the Indian 2011 census (the DHS sampling frame) confirms that following DHS methods, the majority of clusters will fall within the range of 100-150 households. All clusters in urban areas are census enumeration blocks which are designed to contain approximately 125 households. In rural areas, cluster size ranges between 40 and 300, but 94% of the Indian rural population lives in villages (the rural PSU unit) of more than 500 individuals^8^ (results not presented for over 300 residents), and such PSUs will be segmented to hold approximately 100-150 households per cluster following DHS sampling procedures. Cluster size, and hence probability of household selection, is therefore similar for the large majority of both the urban and rural population by sampling design, which lessens the importance of adjusting the household level weight for household selection probability when approximating the cluster-level weight.

While the estimates presented in this analysis using these approximated cluster-level weights may contain some imprecision, particularly in environmental variables with associations further from the null, we believe the approximation presented here delivers meaningful estimates in a context in which no other authors have proposed approximation of weighting using the NFHS-5 dataset.

## **Supplementary tables and figures**

**Supplementary Table 1: Characteristics of excluded participants across key demographics presented as a proportion of all surveyed reproductive-age women with a given characteristic.**

|  | **Reason for exclusion** | | |
| --- | --- | --- | --- |
|  | **No anaemia or BMI measurement**  **(n or % excluded)** | **Pregnant or ≤3 months post-partum**  **(n or % excluded)** | **Not de jure household resident**  **(n or % excluded)** |
| N | 35,612 | 44,119 | 18,312 |
| **Education** | |  |  |
| No education | 8128 (5.6) | 7671 (4.5) | 2447 (1.6) |
| Incomplete primary | 3672 (4.9) | 4925 (5.3) | 1760 (2.4) |
| Incomplete secondary | 15958 (5.7) | 23446 (6.5) | 9161 (3.1) |
| Complete secondary or higher | 7854 (8.2) | 8077 (6.3) | 4944 (4.0) |
| p value | <0.001 | <0.001 | <0.001 |
| **Age** |  |  |  |
| 15-19 | 7017 (6.7) | 4193 (3.8) | 2928 (2.7) |
| 20-24 | 5755 (6.1) | 17553 (15.0) | 6170 (6.0) |
| 25-29 | 5145 (5.5) | 14443 (11.4) | 4635 (4.4) |
| 30-34 | 4337 (5.5) | 5636 (4.9) | 2202 (2.5) |
| 35-39 | 4606 (5.9) | 1876 (1.4) | 1129 (1.2) |
| 40-44 | 4147 (6.1) | 352 (0.3) | 647 (0.8) |
| 45-49 | 4605 (6.5) | 66 (0.1) | 601 (0.8) |
| p value | 0.21 | <0.001 | <0.001 |
| **Wealth quintile** | |  |  |
| Poorest | 5986 (5.0) | 9741 (6.9) | 3408 (3.1) |
| Poorer | 6653 (5.4) | 9498 (6.0) | 3708 (2.9) |
| Middle | 6734 (5.4) | 8835 (5.8) | 3762 (2.8) |
| Richer | 7125 (6.3) | 8192 (5.4) | 3567 (2.7) |
| Richest | 9114 (8.1) | 7853 (5.3) | 3867 (2.8) |
| p value | <0.001 | <0.001 | 0.002 |
| **Residence** | |  |  |
| Urban | 12763 (8.9) | 8453 (4.5) | 3658 (2.2) |
| Rural | 22849 (4.7) | 35666 (6.5) | 14654 (3.2) |
| p value | <0.001 | <0.001 | <0.001 |
| **Region** |  |  |  |
| Northern | 7580 (6.3) | 8051 (5.9) | 3415 (2.9) |
| North Eastern | 3191 (3.6) | 6803 (6.2) | 901 (0.9) |
| Central | 11659 (7.8) | 12056 (6.6) | 5431 (3.1) |
| Eastern | 4806 (4.3) | 8358 (6.8) | 3925 (3.4) |
| Western | 2747 (6.4) | 3594 (4.9) | 1464 (2.2) |
| Southern | 5629 (5.9) | 5257 (4.6) | 3176 (2.7) |
| p value | 0.001 | <0.001 | 0.009 |
| This table presents weighted frequencies (weighted %). Percentages are a weighted row percentage of those with marker for exclusion among all excluded and included women.  P-values presented for adjusted F test.  Notes: This table is for the paper’s main analysis using the Indian National Family Health Survey round 5 (2019-2021) survey. Because some individuals had multiple markers for exclusion, in total 91,524 women were excluded from regression analysis. Individuals with no outcome measure were most commonly either not present at time of measurement or refused consent for anaemia or BMI assessments. A flow diagram for exclusion is presented in Figure 3. Women who weren’t de jure household members were not excluded from prevalence estimates. | | | |

**Supplementary Table 2: Potential risk factors for the double burdens of anaemia with underweight or overweight/obesity derived from single burden frameworks and mapped to available DHS variables in NFHS-5.**

|  |  | Frameworks | | | | | | | | | DHS variables available in NFHS-5 |
| --- | --- | --- | --- | --- | --- | --- | --- | --- | --- | --- | --- |
|  |  | **Under-weight** | | **Overweight/**  **obesity** | | | | **Anaemia** | | |  |
|  | **Risk factors identified in single burden frameworks** | **UNICEF 2020** | **UNICEF 2013** | | **UNICEF 2020** | **Foresight** | **ROOTS** | | **Balarajan** *et al.* | **WHO AAR** |  |
| Proximal  Distal | Inadequate nutrient intake and absorption | X | X | |  |  |  | | X | X | Frequency of iron-rich food consumption |
|  |  | X | X | |  |  |  | | X | X | Vegetarian diet |
|  | Unhealthy food consumption |  |  | | X | X | X | |  |  | Frequency of unhealthy food consumption |
|  | Infectious disease exposure |  | X | |  |  | X | | X | X | Malaria exposure proxied at cluster level |
|  | Chronic disease, inflammation, gynaecological and obstetric conditions |  | X | |  | X | X | |  | X | Has chronic kidney disease |
|  |  |  | X | |  | X | X | |  | X | Has cancer |
|  |  |  | X | |  | X | X | |  | X | Has thyroid disorder |
|  |  |  | X | |  | X | X | |  | X | Has heart disease |
|  |  |  | X | |  | X | X | |  | X | Has diabetes |
|  | Genetic haemoglobin disorders |  |  | |  |  |  | | X | X |  |
|  | Genetic risk of obesity  Depression or stress and associated medicine  Lack of sleep |  |  | |  | X | X | |  |  |  |
|  |  |  |  | |  | X | X | |  |  |  |
|  |  |  |  | |  |  | X | |  |  |  |
|  | Physical activity |  |  | |  | X |  | |  |  |  |
|  | Alcohol consumption |  |  | |  | X |  | |  |  | Drinks alcohol |
| Intermediate | Household food insecurity | X | X | |  |  |  | |  | X | BPL card (entitles household to subsidised food grain) |
|  | Poor quality and diversity of diets | X |  | |  |  |  | | X | X |  |
|  | Access to food fortification or supplementation |  |  | |  |  |  | | X |  | During pregnancy, given or bought iron tablets |
|  | Access to health and nutrition services and interventions | X |  | | X |  |  | |  | X | Difficulty seeking medical help: no providers at health centre |
|  |  |  |  | |  |  |  | |  |  | Difficulty seeking medical help: no drugs at health centre |
|  |  |  |  | |  |  |  | |  |  | Difficulty seeking medical help: distance to health centre |
|  |  |  |  | |  |  |  | |  |  | Covered by health insurance |
|  |  |  |  | |  |  |  | |  |  | Received nutrition counselling during last pregnancy |
|  | Access to water, sanitation and hygiene services | X |  | |  |  |  | | X | X | Improved source of drinking water |
|  |  | X |  | |  | | | | X | X | Improved toilet facility |
|  | Access to family planning and maternal services |  |  | |  |  |  | |  | X | Current use of modern family planning method |
|  |  |  |  | |  |  |  | |  | X | 4 or more antenatal care visits during pregnancy for last birth |
|  |  |  |  | |  |  |  | |  | X | Delivered last birth in a health facility |
|  |  |  |  | |  |  |  | |  | X | Took deworming prophylaxis during last pregnancy |
|  | Environments that promote physical activity |  |  | | X | X |  | |  |  |  |
|  | Healthy food environment | X | X | | X | X | X | |  |  |  |
|  | Passive entertainment options |  |  | |  | X |  | |  |  |  |
| Distal | Education | X | X | | X |  |  | | X | X | Educational attainment |
|  | Household employment |  | X | |  | X |  | |  |  | Respondent currently employed |
|  | Poverty | X | X | | X | X |  | | X | X | Household wealth index |
|  | Cultural norms and behaviours | X | X | | X | X |  | | X | X | Marital status |
|  |  |  |  | |  |  |  | |  |  | Caste |
|  |  |  |  | |  |  |  | |  |  | Religion |
|  |  |  |  | |  |  |  | |  |  | Number of household members |
|  |  |  |  | |  |  |  | |  |  | Attitudes to wife-beating |
|  |  |  |  | |  |  |  | |  |  | Women’s participation in decision-making |
|  | Health policies | X |  | | X |  |  | |  | X |  |
|  | Physiological vulnerability due to fertility factors |  |  | |  |  | X | | X |  | Total children ever born (gravidity) |
|  |  |  |  | |  |  |  | |  |  | Short inter-birth intervals (<24 months) |
|  |  |  |  | |  |  |  | |  |  | Adolescent pregnancy (<20 years) |
|  | Conflict |  |  | |  |  |  | |  | X |  |
|  | Household access to adequate land |  | X | |  |  |  | |  |  | Household owns land usable for agriculture |
| Fundamental | Ecology, climate and geography | X |  | | X | X |  | | X |  | Region |
|  |  |  |  | |  |  |  | |  |  | Urban/rural residence |
|  |  |  |  | |  |  |  | |  |  | Climate (cluster-level variables mapped from external data sources) |
|  | Political economy | X | X | | X | X |  | | X | X |  |
|  | Inequity |  |  | |  |  |  | |  | X |  |
| Further factors identified from literature review of double burden determinants | | | | | | | | | | | |
|  | Age |  |  | |  |  |  | |  |  | Age |
|  | Tobacco consumption |  |  | |  |  |  | |  |  | Uses tobacco |
| Notes: Underlined DHS variables are available in the NFHS-5 and other DHS surveys, but are not available for all women of reproductive age in the NFHS-5, and as such are not carried forward to the conceptual framework.  WRA = women of reproductive age (15-49). DHS geospatial covariate include the °Cluster level’ ecological variables identified in the table. These are variables derived from diverse remote sensing sources and which have been mapped to within 10km of the sampling clusters used in the DHS round.  Frameworks: UNICEF 2020: UNICEF 2020 framework for Maternal and Child Nutrition^1^; UNICEF 2013: UNICEF 2013 framework for Maternal and Child Undernutrition^2^; Foresights: Foresight 2007 Obesity System Map^3^; ROOTS: ROOTS obesity framework^4^; Balarajan et al., 2011^5^; WHO ARR: WHO accelerating anaemia reduction^6^. | | | | | | | | | | | |

**Supplementary Table 3: Cutoffs used for categorisation of BMI for adolescents based on a projected BMI at age 18 corresponding to Asian population BMI categorisation (Underweight: <18.5 kg/m^2^; Overweight/obesity: ≥ 23 kg/m^2^) using the International Obesity Taskforce growth reference curve.**

|  | **BMI classification (kg/m^2^)** | | |
| --- | --- | --- | --- |
| **Age** | **Underweight** | **Normal weight** | **Overweight/**  **obesity** |
| 15 | <17.43 | 17.43 ≤ BMI < 21.88 | ≥ 21.88 |
| 16 | <17.90 | 17.90 ≤ BMI < 22.35 | ≥ 22.35 |
| 17 | <18.24 | 18.24 ≤ BMI < 22.70 | ≥ 22.70 |
| *Notes: BMI = Body Mass Index.* | | | |

**Supplementary Table 4: Definition and derivation of all variables included in analysis, derived from the NFHS-5 2019-21 or the DHS geospatial covariates dataset for India 2020.**

| Variable name | Form | Derivation |
| --- | --- | --- |
| Iron-rich food consumption index | Continuous | An index from 0 to 10 based on self-reported frequency of consumption in a typical month of food from the following five categories: dark green leafy vegetables, pulses or beans, fish, chicken or meat, or egg. Daily consumption was valued at 2 points while weekly consumption was valued at 1 point on the index. |
| Vegetarian | Binary | Whether or not the woman is a lacto-vegetarian based on self-reporting never consuming egg, chicken/meat, or fish. |
| Daily unhealthy food consumption | Binary | Whether or not an individual self-reports consuming either fried food or aerated drinks daily |
| Drinks alcohol | Binary | Whether or not an individual self-reports ever drinking alcohol |
| Uses tobacco | Binary | Whether or not an individual self-reports consuming tobacco in any form |
| Has health insurance | Binary | Whether or not the woman is covered by any private or government health insurance |
| Difficulty with distance to health facility | Binary | Whether or not an individual considers the distance to a health facility to be ‘a big problem for accessing healthcare when she is sick |
| Concern no providers or drugs at health facility | Binary | Whether or not an individual considers there either being no drugs or no providers available at the health facility to be ‘a big problem’ for accessing healthcare when she is sick |
| Modern contraceptive use | Binary | Whether or not the woman is currently using the pill, female or male sterilization, IUD, injectables, implants, male or female condom, diaphragm, or emergency contraception |
| Unimproved drinking water | Binary | Whether or not an individual resides in a household with an unimproved drinking water source, as defined by the DHS, which include surface water or unprotected wells or springs. |
| Unimproved toilet facility | Binary | Whether or not an individual resides in a household with an unimproved toilet facility, as defined by the DHS, which include open defecation or a pit latrine without a slab. |
| Has BPL card | Binary | Whether or not the household is in possession of a Below Poverty Line card (which entitles the household to subsided food rations). |
| Age | Categorical | Five categories of age in five-year intervals |
| Residence | Binary | Whether the individual was interviewed in a cluster identified as urban or rural by the DHS |
| Education | Categorical | Four categories of educational attainment |
| Wealth quintile | Categorical | Quintiles of the DHS wealth index. The DHS constructs a wealth index using principal component analysis of household items, assets, sanitation facilities and house construction materials. |
| Social group | Categorical | Four categories for membership of a caste or tribe designated by the government as disadvantaged and usually eligible for affirmative action. |
| Number of household members | Categorical | Three categories selected based on the median value to represent fewer than average (1-3 members), average (4-5 members), and more than average (6+ members) |
| Household owns agricultural land | Binary | Whether or not the household owns land that can be used for agriculture |
| Marital status | Categorical | Three categories of never marriage (or union), current marriage or past marriage. |
| Region | Categorical | Six categories for the six geographical regions of India, based on state’s membership of zonal councils. |
| Religion | Categorical | Three categories of religions with over 5% prevalence at the national level and an ‘other’ category |
| Adolescent birth (<20 years) | Binary | Whether or not the woman had at least one birth before the age of 20. Adolescents who have not yet reached age 20 are considered not exposed to adolescent birth even if they may go on to be. |
| Short birth interval (<24 months) | Binary | Whether or not any of the intervals between a woman’s births are shorter than 24 months. Individuals with a gravidity of one or zero are considered not exposed to short birth intervals even if they may go on to be. |
| Gravidity | Continuous | Number of children ever born to the woman |
| Change in number of hot days | Categorical | Three categories of the average number of change in average annual number of hot days (defined as >30°C in the Himalayan mountainous region and 40°C elsewhere, an approximation of the IMD’s definition^9^) for the 2010-2020 period versus 1950-2000 period. The IMD definition also specifies a threshold of 37°C for heat waves in coastal regions, which was not included in this definition due to variable non-availability in the dataset used. |
| Change in precipitation intensity | Categorical | Four categories of change in average intensity of precipitation (mm) in the 5% wettest days in the year, for the 2010-2020 period versus 1950-2000 period. The variable is categorised as much less precipitation in the recent period (< -200mm difference), less precipitation (-200mm to -100mm difference), little change (-100mm to 100mm difference) or more precipitation (>100mm difference). |
| SPEI (drought) index | Categorical | Three tertiles of average annual SPEI (drought) index 2010-2020. The first tertile includes clusters with values from -0.03 to -0.18. The second tertile includes clusters with values from -0.18 to -0.21. The third tertiles includes clusters with values from -0.21 to -0.34. A change of 0.50 units on the SPEI scale is generally considered to represent a change in climatic type (e.g. from mild to moderate drought). |
| Malaria incidence | Categorical | Three categories of Plasmodium Falciparum malaria incidence in 2020 (clinical cases) categorised according to the WHO Global Observatory definition of low (<1 annual clinical case per thousand population), moderate (1-10 cases per thousand population) and high (>10 cases per thousand population) transmission zones. The data are derived from the DHS geospatial covariates dataset which uses data from the Malaria Atlas Project.^10^ |

#### Note on environmental variables

The international anaemia, overweight/obesity and underweight frameworks commonly included climate, ecology and environment as determinants of malnutrition. In particular, we investigated how changing environmental factors may be associated with outcomes. The World Bank identifies heat waves, drought and cyclones and other causes of flooding as the major climate risks facing India.^11^

Heat waves are proxied through the average number of hot days in a year, approximating the India Meteorological Department’s (IMD) definition of a heatwave,^9^ without making a distinction for coastal areas due to lack of data availability. Flooding risk in India is complex, but one driver is unusually heavy precipitation, especially following dry spells. Intensity of precipitation is proxied by average precipitation (centimetres) in the 5% wettest days annually. Drought risk is proxied using the Standardised Precipitation Evapotranspiration Index (SPEI) index value, a measure of the integrated water deficit in a location compared to a long-term average, taking into account the contribution of temperature-dependent evapotranspiration. Changes in each of the average annual number of hot days and average annual precipitation intensity, between a baseline of 1950-2000 and the recent 2010-2020 period, are calculated to form our environmental anomaly variables. SPEI (drought) index inherently includes a comparison to the long-term average in that cluster, so the annual average value for the 2010-2020 period is taken without further comparison to the 1950-2000 average.

Women included in prevalence estimates: (n = 646,210)

Not a ‘de jure’ household member (n = 13,619)

Women not interviewed: (n = 23,061)

Women identified by DHS as eligible for interview during household visit: (n = 747,176)

**Survey**

Women’s individual recode sample: (n = 724,115)

Total (n = 77,905)*

No outcome measure:

No BMI measurement: (n = 23,957)

BMI measurement inconsistent: (n = 796)

No anaemia measurement: (n = 33,962)

Pregnant or up to 3 months postpartum: (n = 44,119)

**Exclusion**

Women included in regression analysis: (n = 632,591)

Women in complete-case multivariable analysis: (n = 632, 582)

Survey weighted number of women in complete-case multivariable analysis: (n = 619,592)

**Analysis**

Supplementary Figure 1: Flow diagram of the selection of the survey and study participants from the NFHS-5, 2019-2021.

*Notes: Numbers presented may not sum to the total as some individuals met more than one of the exclusion criteria presented

Women included in prevalence estimates: (n = 637,610)

Not a ‘de jure’ household member (n = 17,581)

Women not interviewed: (n = 24,189)

Women identified by DHS as eligible for interview during household visit: (n = 723,875)

**Survey**

Women’s individual recode sample:

(n = 699,686)

Total (n = 62,076)*

No outcome measure:

No BMI measurement: (n = 11,810)

BMI measurement inconsistent: (n = 720)

No anaemia measurement: (n = 14,775)

Pregnant or up to 3 months postpartum:

(n = 46,778)

**Exclusion**

Women included in regression analysis: (n = 620,029)

Women in complete-case multivariable analysis:

(n = 620,029)

Survey weighted number of women in complete-case multivariable analysis:

(n = 615,684)

**Analysis**

Supplementary Figure 2: Flow diagram of the selection of the survey and study participants from the NFHS-4, 2015-2016.

*Notes: Numbers presented may not sum to the total as some individuals met more than one of the exclusion criteria presented

Women included in prevalence estimates: (n = 104,609)

Not a ‘de jure’ household member (n = 4,014)

Women not interviewed: (n = 7,211)

Women identified by DHS as eligible for interview during household visit: (n = 131,596)

**Survey**

Women’s individual recode sample:

(n = 124,385)

Total (n = 16,863)*

No outcome measure:

No BMI measurement: (n = 5.604)

BMI measurement inconsistent: (n = 47)

No anaemia measurement: (n = 11,671)

Pregnant or up to 3 months postpartum:

(n = 8,835)

**Exclusion**

Women included in regression analysis: (n = 100,595)

Women in complete-case multivariable analysis:

(n = 99,794)

Survey weighted number of women in complete-case multivariable analysis:

(n = 102,058)

**Analysis**

Supplementary Figure 3: Flow diagram of the selection of the survey and study participants from the NFHS-3, 2005-2006.

*Notes: Numbers presented may not sum to the total as some individuals met more than one of the exclusion criteria presented

Supplementary Table 5: Sensitivity analysis for the associations of potential risk factors with the single and double burdens of malnutrition compared to no malnutrition burden, using moderate/severe threshold for anaemia NFHS-5, 2019-2021.

|  | **Single burdens** | | | **Double burdens** | | |
| --- | --- | --- | --- | --- | --- | --- |
|  | **OR (95% CI)** | | | **OR (95% CI)** | | |
|  | **Moderate/severe anaemia only** | **Underweight only** | **Overweight/** 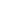 **obesity only** | | **Underweight and moderate/severe anaemia** | **Overweight/**  **obesity and moderate/severe anaemia** |
| ***N =*** | 98,437 | 65,547 | 166,209 | | 42,732 | 67,913 |
|  | | | | | | |
| ***Proximal factors (individual level)*** | |  |  | |  |  |
| **Iron-rich food consumption** **index** | *** | *** | *** | | *** |  |
|  | 0.99 (0.98, 0.99) | 0.98 (0.98, 0.99) | 1.02 (1.01, 1.03) | | 0.97 (0.96, 0.98) | 1.02 (1.00, 1.03) |
| **Vegetarian** |  |  |  | |  | *** |
| No | 1.00 (ref) | 1.00 (ref) | 1.00 (ref) | | 1.00 (ref) | 1.00 (ref) |
| Yes | 1.04 (1.00, 1.07) | 1.01 (0.98, 1.04) | 1.00 (0.97, 1.03) | | 1.07 (1.02, 1.11) | 1.10 (1.06, 1.15) |
| **Daily unhealthy food consumption** |  |  |  | |  |  |
| No | 1.00 (ref) | 1.00 (ref) | 1.00 (ref) | | 1.00 (ref) | 1.00 (ref) |
| Yes | 1.04 (0.99, 1.08) | 1.01 (0.96, 1.07) | 1.04 (1.00, 1.08) | | 0.97 (0.92, 1.03) | 1.00 (0.95, 1.06) |
| **Drinks alcohol** |  |  |  | |  |  |
| No | 1.00 (ref) | 1.00 (ref) | 1.00 (ref) | | 1.00 (ref) | 1.00 (ref) |
| Yes | 0.95 (0.86, 1.05) | 1.00 (0.88, 1.14) | 0.97 (0.87, 1.07) | | 1.04 (0.92, 1.18) | 0.91 (0.79, 1.05) |
| **Uses tobacco** | *** | *** | *** | | *** |  |
| No | 1.00 (ref) | 1.00 (ref) | 1.00 (ref) | | 1.00 (ref) | 1.00 (ref) |
| Yes | 1.16 (1.10, 1.23) | 1.38 (1.30, 1.47) | 0.86 (0.82, 0.91) | | 1.74 (1.63, 1.86) | 1.00 (0.93, 1.07) |
|  | |  |  | |  |  |
| **Has health insurance** |  |  | *** | |  | *** |
| No | 1.00 (ref) | 1.00 (ref) | 1.00 (ref) | | 1.00 (ref) | 1.00 (ref) |
| Yes | 1.05 (1.01, 1.08) | 1.02 (0.99, 1.05) | 0.93 (0.90, 0.95) | | 1.04 (1.00, 1.09) | 0.94 (0.90, 0.97) |
| **Difficulty with distance to health facility** |  |  |  | |  |  |
| Not a big concern | 1.00 (ref) | 1.00 (ref) | 1.00 (ref) | | 1.00 (ref) | 1.00 (ref) |
| Big concern | 1.03 (1.00, 1.06) | 1.04 (1.00, 1.07) | 0.97 (0.94, 1.00) | | 1.04 (1.00, 1.09) | 1.02 (0.98, 1.06) |
| **Concern no providers or drugs at health facility** |  |  |  | | *** |  |
| Not a big concern | 1.00 (ref) | 1.00 (ref) | 1.00 (ref) | | 1.00 (ref) | 1.00 (ref) |
| Big concern | 1.04 (1.01, 1.07) | 1.02 (0.99, 1.05) | 0.99 (0.97, 1.02) | | 1.06 (1.02, 1.10) | 1.03 (0.99, 1.06) |
| **Modern contraceptive use** |  | *** | *** | | *** |  |
| No | 1.00 (ref) | 1.00 (ref) | 1.00 (ref) | | 1.00 (ref) | 1.00 (ref) |
| Yes | 1.01 (0.98, 1.04) | 0.90 (0.87, 0.93) | 0.93 (0.91, 0.95) | | 0.88 (0.84, 0.91) | 0.96 (0.92, 0.99) |
| **Unimproved drinking water** | *** |  |  | |  |  |
| No | 1.00 (ref) | 1.00 (ref) | 1.00 (ref) | | 1.00 (ref) | 1.00 (ref) |
| Yes (unimproved) | 1.09 (1.04, 1.16) | 1.01 (0.96, 1.07) | 1.00 (0.95, 1.06) | | 1.01 (0.94, 1.08) | 0.93 (0.86, 1.01) |
| **Unimproved toilet facility** |  |  | *** | | *** | *** |
| No | 1.00 (ref) | 1.00 (ref) | 1.00 (ref) | | 1.00 (ref) | 1.00 (ref) |
| Yes (unimproved) | 1.03 (1.00, 1.06) | 1.03 (1.00, 1.07) | 0.93 (0.90, 0.96) | | 1.09 (1.05, 1.14) | 0.91 (0.87, 0.96) |
| **Has BPL card** |  |  | *** | | *** |  |
| No | 1.00 (ref) | 1.00 (ref) | 1.00 (ref) | | 1.00 (ref) | 1.00 (ref) |
| Yes | 1.03 (1.00, 1.06) | 1.01 (0.98, 1.04) | 0.90 (0.88, 0.93) | | 1.08 (1.04, 1.12) | 0.96 (0.93, 1.00) |
| ***Distal factors (individual level)*** |  |  |  | |  |  |
| ***Age*** | *** | *** | *** | | *** | *** |
| 15-19 | 1.07 (1.02, 1.13) | 1.54 (1.46, 1.63) | 0.44 (0.41, 0.46) | | 1.47 (1.37, 1.57) | 0.52 (0.48, 0.56) |
| 20-24 | 0.99 (0.94, 1.03) | 1.35 (1.29, 1.42) | 0.62 (0.60, 0.65) | | 1.28 (1.20, 1.36) | 0.62 (0.58, 0.66) |
| 25-29 | 1.00 (ref) | 1.00 (ref) | 1.00 (ref) | | 1.00 (ref) | 1.00 (ref) |
| 30-35 | 1.00 (0.95, 1.04) | 0.79 (0.76, 0.84) | 1.51 (1.46, 1.57) | | 0.80 (0.75, 0.85) | 1.53 (1.45, 1.62) |
| 35-39 | 0.99 (0.95, 1.04) | 0.67 (0.64, 0.71) | 1.82 (1.75, 1.89) | | 0.72 (0.68, 0.77) | 1.96 (1.85, 2.07) |
| 40-45 | 1.10 (1.04, 1.15) | 0.64 (0.60, 0.68) | 2.12 (2.03, 2.21) | | 0.70 (0.65, 0.75) | 2.49 (2.35, 2.64) |
| 45-49 | 1.01 (0.96, 1.06) | 0.64 (0.60, 0.68) | 2.22 (2.13, 2.32) | | 0.67 (0.62, 0.72) | 2.43 (2.30, 2.58) |
| **Residence** |  | *** | *** | |  | *** |
| Urban residence | 1.00 (ref) | 1.00 (ref) | 1.00 (ref) | | 1.00 (ref) | 1.00 (ref) |
| Rural residence | 0.99 (0.95, 1.04) | 1.09 (1.04, 1.14) | 0.91 (0.88, 0.95) | | 1.08 (1.01, 1.16) | 0.91 (0.86, 0.95) |
| **Education** | *** | *** | *** | | *** | *** |
| No education | 1.00 (ref) | 1.00 (ref) | 1.00 (ref) | | 1.00 (ref) | 1.00 (ref) |
| Incomplete primary | 1.01 (0.97, 1.05) | 0.86 (0.82, 0.90) | 1.19 (1.15, 1.24) | | 0.86 (0.82, 0.91) | 1.24 (1.18, 1.30) |
| Incomplete secondary | 0.98 (0.95, 1.02) | 0.82 (0.79, 0.86) | 1.32 (1.28, 1.36) | | 0.79 (0.75, 0.83) | 1.41 (1.35, 1.48) |
| Complete secondary and higher | 0.85 (0.81, 0.90) | 0.80 (0.76, 0.84) | 1.31 (1.25, 1.37) | | 0.67 (0.63, 0.72) | 1.26 (1.18, 1.34) |
| **Wealth quintile** | *** | *** | *** | | *** | *** |
| Poorest | 1.18 (1.11, 1.26) | 1.76 (1.65, 1.88) | 0.40 (0.38, 0.42) | | 1.87 (1.71, 2.05) | 0.39 (0.36, 0.42) |
| Poorer | 1.13 (1.07, 1.19) | 1.52 (1.43, 1.61) | 0.52 (0.50, 0.55) | | 1.56 (1.43, 1.69) | 0.53 (0.50, 0.57) |
| Middle | 1.17 (1.11, 1.23) | 1.35 (1.28, 1.43) | 0.67 (0.64, 0.70) | | 1.33 (1.22, 1.44) | 0.67 (0.63, 0.71) |
| Richer | 1.10 (1.05, 1.16) | 1.19 (1.12, 1.25) | 0.79 (0.77, 0.83) | | 1.21 (1.12, 1.30) | 0.83 (0.79, 0.87) |
| Richest | 1.00 (ref) | 1.00 (ref) | 1.00 (ref) | | 1.00 (ref) | 1.00 (ref) |
| **Social group** | *** | *** | *** | | *** | *** |
| Scheduled caste | 1.11 (1.06, 1.15) | 1.05 (1.00, 1.10) | 0.88 (0.85, 0.92) | | 1.16 (1.10, 1.24) | 0.97 (0.92, 1.02) |
| Scheduled tribe | 1.33 (1.26, 1.40) | 1.01 (0.96, 1.07) | 0.77 (0.74, 0.81) | | 1.40 (1.31, 1.50) | 0.98 (0.92, 1.05) |
| OBC | 1.00 (0.96, 1.04) | 1.08 (1.04, 1.12) | 0.91 (0.88, 0.94) | | 1.07 (1.01, 1.13) | 0.86 (0.82, 0.90) |
| Other caste, no caste or unsure | 1.00 (ref) | 1.00 (ref) | 1.00 (ref) | | 1.00 (ref) | 1.00 (ref) |
| **Number of household members** |  | *** | *** | | *** |  |
| 3 or fewer | 1.00 (ref) | 1.00 (ref) | 1.00 (ref) | | 1.00 (ref) | 1.00 (ref) |
| 4 or 5 | 1.04 (1.01, 1.08) | 1.11 (1.06, 1.16) | 0.97 (0.94, 1.00) | | 1.13 (1.08, 1.19) | 0.94 (0.90, 0.98) |
| 6 or more | 1.04 (1.00, 1.08) | 1.17 (1.12, 1.23) | 0.93 (0.89, 0.96) | | 1.18 (1.12, 1.25) | 0.92 (0.88, 0.97) |
| **Household owns agricultural land** | *** |  | *** | |  |  |
| No | 1.00 (ref) | 1.00 (ref) | 1.00 (ref) | | 1.00 (ref) | 1.00 (ref) |
| Yes | 1.05 (1.02, 1.08) | 1.04 (1.01, 1.07) | 0.93 (0.90, 0.95) | | 1.02 (0.98, 1.06) | 0.96 (0.92, 0.99) |
| **Marital Status** | *** | *** | *** | |  | *** |
| Never in union | 0.94 (0.89, 0.98) | 1.22 (1.17, 1.28) | 0.65 (0.62, 0.68) | | 1.04 (0.98, 1.11) | 0.58 (0.54, 0.62) |
| Married | 1.00 (ref) | 1.00 (ref) | 1.00 (ref) | | 1.00 (ref) | 1.00 (ref) |
| Divorced/separated/ widowed | 1.12 (1.05, 1.19) | 1.02 (0.94, 1.10) | 0.86 (0.81, 0.90) | | 1.07 (0.99, 1.16) | 0.93 (0.86, 0.99) |
| **Region** | *** | *** | *** | | *** | *** |
| Northern | 1.00 (ref) | 1.00 (ref) | 1.00 (ref) | | 1.00 (ref) | 1.00 (ref) |
| North Eastern | 0.86 (0.81, 0.92) | 0.72 (0.67, 0.78) | 0.86 (0.81, 0.92) | | 0.76 (0.68, 0.83) | 0.83 (0.76, 0.91) |
| Central | 0.73 (0.70, 0.77) | 0.97 (0.92, 1.02) | 1.01 (0.97, 1.05) | | 0.76 (0.71, 0.80) | 0.75 (0.71, 0.79) |
| Eastern | 1.06 (1.00, 1.12) | 1.03 (0.97, 1.09) | 0.97 (0.92, 1.03) | | 1.10 (1.02, 1.18) | 1.19 (1.11, 1.28) |
| Western | 1.06 (0.99, 1.14) | 1.62 (1.52, 1.73) | 0.89 (0.84, 0.95) | | 1.85 (1.67, 2.05) | 0.94 (0.86, 1.03) |
| Southern | 0.88 (0.83, 0.94) | 1.29 (1.20, 1.38) | 1.46 (1.38, 1.55) | | 1.22 (1.12, 1.32) | 1.39 (1.29, 1.51) |
| **Religion** |  | *** | *** | | *** |  |
| Hindu | 1.00 (ref) | 1.00 (ref) | 1.00 (ref) | | 1.00 (ref) | 1.00 (ref) |
| Muslim | 0.98 (0.93, 1.02) | 0.90 (0.86, 0.95) | 1.16 (1.11, 1.21) | | 0.86 (0.80, 0.92) | 1.10 (1.04, 1.17) |
| Other | 0.88 (0.82, 0.94) | 0.87 (0.81, 0.93) | 1.20 (1.13, 1.27) | | 0.79 (0.73, 0.87) | 1.07 (0.98, 1.16) |
| **Adolescent birth (<20 years)** |  | *** | *** | |  | *** |
| No | 1.00 (ref) | 1.00 (ref) | 1.00 (ref) | | 1.00 (ref) | 1.00 (ref) |
| Yes | 1.00 (0.97, 1.03) | 0.95 (0.91, 0.98) | 1.07 (1.05, 1.10) | | 0.95 (0.91, 0.99) | 1.10 (1.07, 1.14) |
| **Short birth interval (<24 months)** |  |  | *** | |  | *** |
| No | 1.00 (ref) | 1.00 (ref) | 1.00 (ref) | | 1.00 (ref) | 1.00 (ref) |
| Yes | 0.99 (0.96, 1.02) | 1.05 (1.01, 1.09) | 1.07 (1.04, 1.10) | | 0.96 (0.92, 1.01) | 1.07 (1.03, 1.11) |
|  | |  |  | |  |  |
| **Change in annual number of hot days** | *** | *** | *** | |  | *** |
| Little change (<5 days more than average) | 1.00 (ref) | 1.00 (ref) | 1.00 (ref) | | 1.00 (ref) | 1.00 (ref) |
| More hot days (5 to 10 days more than average) | 0.88 (0.85, 0.91) | 1.10 (1.06, 1.14) | 0.92 (0.89, 0.95) | | 0.98 (0.94, 1.03) | 0.84 (0.80, 0.88) |
| Many more hot days (>10 days more than average) | 0.91 (0.87, 0.95) | 1.02 (0.98, 1.07) | 0.82 (0.79, 0.85) | | 0.96 (0.90, 1.02) | 0.76 (0.72, 0.80) |
| **Change in annual precipitation intensity** |  |  |  | |  |  |
| Much less precipitation (< -200mm less than average) | 0.92 (0.83, 1.02) | 0.94 (0.83, 1.05) | 1.14 (1.02, 1.27) | | 0.84 (0.71, 0.99) | 1.11 (0.95, 1.29) |
| Less precipitation (-100mm to -200mm less than average) | 1.04 (0.99, 1.09) | 1.01 (0.96, 1.06) | 1.00 (0.96, 1.05) | | 0.99 (0.92, 1.06) | 1.10 (1.03, 1.17) |
| Little change (-100mm to +100mm change) | 1.00 (ref) | 1.00 (ref) | 1.00 (ref) | | 1.00 (ref) | 1.00 (ref) |
| More precipitation (> 100mm more than average) | 1.03 (0.97, 1.08) | 0.97 (0.92, 1.02) | 0.99 (0.95, 1.04) | | 1.04 (0.97, 1.12) | 0.96 (0.91, 1.03) |
| **SPEI (drought) index** | *** |  | *** | | *** | *** |
| First tertile (little change) | 1.00 (ref) | 1.00 (ref) | 1.00 (ref) | | 1.00 (ref) | 1.00 (ref) |
| Second tertile (drier than average) | 1.02 (0.98, 1.06) | 1.02 (0.98, 1.06) | 0.98 (0.95, 1.02) | | 1.04 (0.99, 1.09) | 0.97 (0.92, 1.02) |
| Third tertile (drier than average) | 1.12 (1.07, 1.17) | 1.06 (1.01, 1.11) | 1.08 (1.03, 1.13) | | 1.13 (1.06, 1.21) | 1.15 (1.08, 1.23) |
| **Malaria incidence** |  | *** | *** | | *** |  |
| Low transmission | 1.00 (ref) | 1.00 (ref) | 1.00 (ref) | | 1.00 (ref) | 1.00 (ref) |
| Moderate transmission | 1.03 (0.98, 1.08) | 1.04 (0.99, 1.09) | 0.93 (0.88, 0.98) | | 1.07 (1.00, 1.14) | 0.92 (0.86, 0.99) |
| High transmission | 1.09 (1.04, 1.14) | 1.13 (1.08, 1.19) | 0.93 (0.88, 0.97) | | 1.22 (1.15, 1.30) | 0.96 (0.90, 1.03) |
|  |  |  |  | |  |  |
| *** indicates significant joint Wald test at the Bonferroni-corrected threshold (p <0.00179).  Notes: This table presents odds ratios and 95% confidence intervals from a multilevel multinomial logistic regression model. The baseline is normal weight without anaemia (no burden of malnutrition) in all outcomes (N = 191,753). Pregnant women and those up to 3 months post-partum were excluded from analysis. Counts presented are unweighted. Moderate/severe anaemia: haemoglobin concentration <11.0g/dL; Underweight: BMI <18.5kg/m^2^; Overweight/obesity: BMI >23kg/m^2^. BMI: Body Mass Index. BPL card = Below Poverty Line card (entitles household to subsidised food rations). | | | | | | |

## References

1 UNICEF. UNICEF Conceptual Framework on the Determinants of Maternal and Child Nutrition. 2020. https://www.unicef.org/documents/conceptual-framework-nutrition (accessed May 26, 2024).

2 UNICEF. Improving child nutrition: The achievable imperative for global progress. New York: UNICEF HQ, 2013.

3 UK Government’s Foresight Programme. Tackling obesities: future choices - full systems map. 2007.

4 World Obesity Federation. The ROOTS of Obesity. 2020. https://www.worldobesity.org/what-we-do/our-policy-priorities/the-roots-of-obesity (accessed June 13, 2024).

5 Balarajan Y, Ramakrishnan U, Özaltin E, Shankar AH, Subramanian SV. Anaemia in low-income and middle-income countries. *The Lancet* 2011; **378**: 2123–35.

6 World Health Organisation. Accelerating anaemia reduction: a comprehensive framework for action. Geneva, 2023 https://www.who.int/publications/i/item/9789240074033 (accessed June 13, 2024).

7 #29430: : India » Construction of level weights from individual weights. The DHS Program User Forum. 2024; published online June 17. https://userforum.dhsprogram.com/index.php?t=msg&goto=24938&&srch=india+multilevel#msg_24938 (accessed June 22, 2024).

8 Office of the Registrar General & Census Commissioner, India. PCA STATEMENT 1: Percentage of population living in villages of various population size with reference to the total rural population : 2011. https://censusindia.gov.in/census.website/data/census-tables (accessed June 22, 2024).

9 India Meteorological Department. Meteorological Monograph, Heat and Cold Waves in India: Chapter 2 - Cold and Heat Wave Indices and Methodology. 2023.

10 Malaria Atlas Project. Plasmodium falciparum incidence version 2020. 2020.

11 World Bank Group. Climate Risk Country Profile: India. 2021.
